# Patient‐ and Clinician‐Reported Outcomes and Outcome Measures Evaluating Timing of Implant Placement in the Edentulous Maxilla: A Systematic Review of Clinical Studies

**DOI:** 10.1111/clr.14454

**Published:** 2026-02-24

**Authors:** Giuseppe A. Romito, Isabella Neme Ribeiro dos Reis, Mohamed A. Hassan, Cristina Cunha Villar, Helena Francisco, Claudio Mendes Pannutti

**Affiliations:** ^1^ Division of Periodontology, Department of Stomatology, School of Dentistry University of São Paulo São Paulo Brazil; ^2^ Dental Research Division, Department of Periodontology and Oral Implantology Guarulhos University – UNG Guarulhos Brazil; ^3^ Department of Oral Surgery and Implant Dentistry, Faculty of Dental Medicine Lisbon University Lisbon Portugal; ^4^ Implantology Institute Lisbon Portugal

**Keywords:** dental implant, edentulous, maxilla, outcome reporting

## Abstract

**Aim:**

To address what patient‐reported outcomes (PROs) and clinician‐reported outcomes (ClinROs) have been reported, and what measures (PROMs and CROMs) have been used to assess them in clinical studies on the timing of implant placement in the edentulous maxilla?

**Materials and Methods:**

Systematic searches were conducted in five databases to identify longitudinal prospective clinical studies. PROMs, CROMs, and methods were extracted. Risk of bias was assessed based on study design, and outcomes were analyzed descriptively.

**Results:**

Thirteen studies were included: 1 randomized controlled trial and 12 case series. Delayed placement was reported in 9 studies, immediate in 2, and both in 2. Regarding PROs, 16 outcomes were identified, including oral health‐related quality of life (reported in 53.85%), pain intensity (38.46%), satisfaction with the prosthesis (23.08%), postoperative drug use (15.38%), and outcomes related to well‐being during surgery, treatment satisfaction, oral health satisfaction, psychosocial impact, daily life impairment and prosthesis functionality, among others (each 7.69%). Eighteen ClinROs were identified. Implant survival was reported in 38.46% of studies, prosthetic complications in 30.77%, and prosthetic survival in 23.08%. Peri‐implant health parameters—bone level changes, probing depth, plaque and bleeding indices—were reported in 23.08%. Fewer studies assessed swelling (15.38%), surgical parameters (11.54%), articulation, and oromyofunctional behavior (5.6%). Assessment methods varied widely, limiting comparability. No clear pattern was observed regarding the timing of implant placement.

**Conclusions:**

PROs and ClinROs exhibited significant heterogeneity in domains, methods, and reporting. Harmonizing outcome selection and establishing a core outcome set are needed to enhance comparability and reliability.

## 1 Introduction

Edentulism negatively impacts a patient's life by impairing psychosocial functioning, nutrition, and overall health and diminishing quality of life (Felton 2009). This challenge is even greater in the maxilla due to the high aesthetic demands (Busenlechner et al. 2016).

Patients with maxillary hopeless residual dentition who are to be rehabilitated with implants and implant‐supported restorations often face difficulties and express concerns about the post‐extraction process (Cordaro et al. 2010) and the management of the provisional phase until their definitive prosthetic restoration (Busenlechner et al. 2016). One crucial decision in this context is the timing of implant placement after teeth extraction.

The traditional two‐stage protocol involves waiting 3–6 months after tooth extraction before implant placement (Brånemark et al. 1977; Adell et al. 1981). To shorten treatment time, early and immediate implant placement protocols have been proposed. Early implant placement occurs 4–8 weeks after extraction, following most soft‐tissue healing but before complete bone maturation. Immediate implant placement takes place at the time of extraction or within 10 days (Tonetti et al. 2019). The choice of the protocol should be made before tooth extraction, based on a careful analysis of factors such as bone volume, the integrity of the socket, the presence of infection, and hard‐ and soft‐tissue defects. Additionally, patient‐specific factors, such as smoking habits, should be considered (Tonetti et al. 2019). High survival and similar failure rates have been observed for implants placed at different times in the fully edentulous maxilla (Covani et al. 2012; Ciabattoni et al. 2017).

Traditional outcome measures, such as marginal bone levels, peri‐implant clinical parameters, and the survival of implants and prostheses, have limitations due to their focus on biological and mechanical aspects, which do not reflect the patient's perspective (De Bruyn et al. 2015). Consequently, the scientific community has recently shifted towards incorporating patient‐reported outcome measures (PROMs) and clinician‐reported outcome measures (CROMs) (Powers et al. 2017; Yu et al. 2023). Patient‐reported outcomes (PROs) refer to the actual health outcomes as experienced by patients, such as pain, quality of life, or satisfaction. PROMs are the structured tools, like questionnaires or scales, that capture these PROs directly from patients, without any alterations or interpretations by clinicians or others, ensuring a standardized assessment of the patient's perspective (Weldring and Smith 2013; FDA Glossary 2018). In contrast, clinician‐reported outcomes (ClinROs) are derived from evaluations made by healthcare professionals based on clinical observations and objective tests, requiring specialized training to accurately interpret signs, behaviors, or other manifestations related to a condition (FDA Glossary 2018). CROMs include reports of specific clinical findings or events and/or the use of rating scales (FDA Glossary 2018).

Recently, the ID‐COSM project established a minimum core outcome set to standardize research in implant dentistry, identifying essential domains for clinical studies in this field. This project, along with its supporting systematic reviews, highlighted four core areas—pathophysiology, lifespan, life impact, and access to care‐health economics—and defined 11 mandatory outcome domains that should be included in the protocol and reporting of clinical trials in implant dentistry (Tonetti et al. 2023). However, while ID‐COSM provides valuable guidance on essential outcomes, particularly traditional ones, it does not specify which PROMs and CROMs should be used in specific clinical scenarios. Additionally, it does not provide guidance on the specific tools, methods of aggregation, analysis metrics, or ideal time points for assessing these outcomes and the interpretation and reporting of results.

There is a growing trend to analyze PROMs and CROMs alongside traditional metrics in implant dentistry for a more comprehensive evaluation (De Bruyn et al. 2015; Messias et al. 2023), and this shift highlights the need for standardizing these outcomes (De Bruyn et al. 2015; Souza et al. 2023; Messias et al. 2023). Consistent methodologies are essential to ensure that findings are comparable and valuable for clinical decision‐making. The rationale for this review is the necessity of harmonization in PROMs and CROMs reporting in clinical studies concerning the timing of implant placement in the edentulous maxilla.

The systematic review aims to identify, categorize, and summarize the PROs, PROMs, ClinROs, CROMs, and methods used in these studies over the past 10 years, allowing for the establishment of consistent outcomes and methodologies for future research in this clinical scenario, which in turn will facilitate the development of a core outcome set.

## 2 Materials and Methods

### 2.1 Protocol and Registration

The protocol for this systematic review was registered at PROSPERO (CRD42024519353). This review was reported in accordance with the guidelines of the Preferred Reporting Items for Systematic Review and Meta‐Analyses (PRISMA 2020; Page et al. 2021). This systematic review was commissioned by the Consensus Committee of the 1st Global Consensus for Clinical Guidelines and conducted in accordance with its established guidelines.

### 2.2 Focused Questions and PICOS Outline

#### 2.2.1 Question 1

(1) In patients in need of full rehabilitation of the maxillary arch with dental implant therapy according to different implant placement timing protocols, what PROs and ClinROs have been reported?

#### 2.2.2 Question 2

(2) In patients in need of full rehabilitation of the maxillary arch with dental implant therapy according to different implant placement timing protocols, what measures PROMs and CROMs have been employed to assess PROs and ClinROs?

Although the present review addresses two focused questions, they explore complementary aspects of the same population, intervention, and outcomes: Question 1 focuses on the identification of reported PROs and ClinROs, while Question 2 addresses the measures used to assess these outcomes (PROs and ClinROs).

#### 2.2.3 PIOS Outline

- **Population (P):** Patients with an edentulous maxilla or those with residual dentition scheduled for extraction and in need of an implant‐supported prosthesis.
- **Intervention (I):** Implant therapy, including immediate implant placement at the time of tooth extraction and subsequent placements at various stages of healing: early soft‐tissue healing [4–8 weeks post‐extraction], partial bone healing [3–4 months post‐extraction], delayed implant placement [more than 4 months post‐extraction] and following alveolar ridge preservation [within 4–8 weeks or 3–4 months after the alveolar ridge preservation procedure].
- **Outcomes (O):** Primary outcomes: PROs/PROMs; Secondary outcomes: ClinROs/CROMs.
- **Study (S):** Prospective interventional and observational studies.

### 2.3 Eligibility Criteria

#### 2.3.1 Inclusion Criteria

- Studies clearly reporting the time of implant placement after tooth extraction, regardless of the presence of a comparative group.
- Prospective interventional and observational studies (e.g., randomized controlled trials, non‐randomized controlled trials, cohort studies, case series with at least 10 cases) to capture a comprehensive range of PROMs and CROMs from both controlled and observational contexts.
- Patients with a completely edentulous maxilla or those scheduled for all maxillary teeth extraction who will receive implants and complete‐arch implant‐supported prostheses.
- Studies evaluating both PROs/PROMs and/or ClinROs/CROMs.
- Studies published within the past 10 years (from 2014 to present) focus on recent practices, in alignment with the guidance provided by the consensus committee that commissioned this review.
- Studies in the English language to promote consistency in data extraction and analysis, as it is the predominant language in this field.

#### 2.3.2 Exclusion Criteria

- Retrospective studies, cross‐sectional studies, case reports, in vitro, and preclinical studies.
- Studies involving implants that had already been placed prior to the study initiation.
- Studies involving implants placed in the pterygoid or zygomatic bones.

### 2.4 Search Methods

The electronic databases searched between April 27 and May 17, 2024, included the Cochrane Central Register of Controlled Trials (CENTRAL), MEDLINE (PubMed), and SCOPUS. Additionally, the bibliographies of the included articles were manually searched. Search results from all databases were then combined, and duplicates were removed. The detailed search strategies are listed in Table S1.

### 2.5 Study Selection and Data Collection

After searching the mentioned electronic databases, the retrieved articles underwent a three‐phase screening process independently conducted by two authors (I.N.R.R. and M.A.H.). Initially, the Rayyan platform (Ouzzani et al. 2016) was used to select titles and abstracts based on the eligibility criteria. Articles that appeared to meet the inclusion criteria or those with insufficient information in the titles and abstracts for a clear decision were chosen for full manuscript evaluation. Subsequently, the full‐text versions of these studies were reviewed. Studies meeting all selection criteria proceeded to data extraction. To ensure consistency, reviewers participated in initial calibration sessions and held periodic meetings after every 100 articles to resolve discrepancies and align methods. Reviewer agreement was measured using the Kappa coefficient. Disagreements were resolved through discussion and consensus, with a third reviewer (C.M.P.) providing arbitration if necessary.

The data extraction included the first author's name, year of publication, country, setting (university or private practice), study design, number of arms, funding, number of patients, sex, mean age, study's maximum follow‐up, primary outcome, timing of implant placement, timing of loading, whether the implants were placed in the maxilla or both the maxilla and mandible, number of implants, number of implants per patient, implant type (e.g., standard implants vs. mini‐dental implants), and type of prosthesis (fixed or removable). For PROs and ClinROs, specific measure (PROMs/CROMs) and methods, interpretation, analysis metrics, method of aggregation, and time points were collected (FDA Glossary 2018; Hopewell et al. 2025).

### 2.6 Risk of Bias

The risk of bias in the included randomized controlled trial was assessed in duplicate by two authors (I.N.R.R. and M.A.H.) as part of the data extraction process, using version 2 of the Cochrane risk‐of‐bias tool (RoB2) for randomized trials (Sterne et al. 2019). Quality assessment of the included case series was done according to the guidelines of the Joanna Briggs Institute (JBI) checklist for case series (Martin 2017).

### 2.7 Data Synthesis

The outcomes for each of the two focused questions were described in detail and analyzed using STATA version 16.1 (StataCorp, College Station, TX, USA). PROMs and CROMs were presented separately. Descriptive statistics, including numbers and percentages, were applied to summarize the frequency distribution of assessed outcomes and measurements. Within each category, similar outcomes were grouped, and tables were constructed to display key information.

## 3 Results

### 3.1 Search and Screening

**FIGURE 1:**
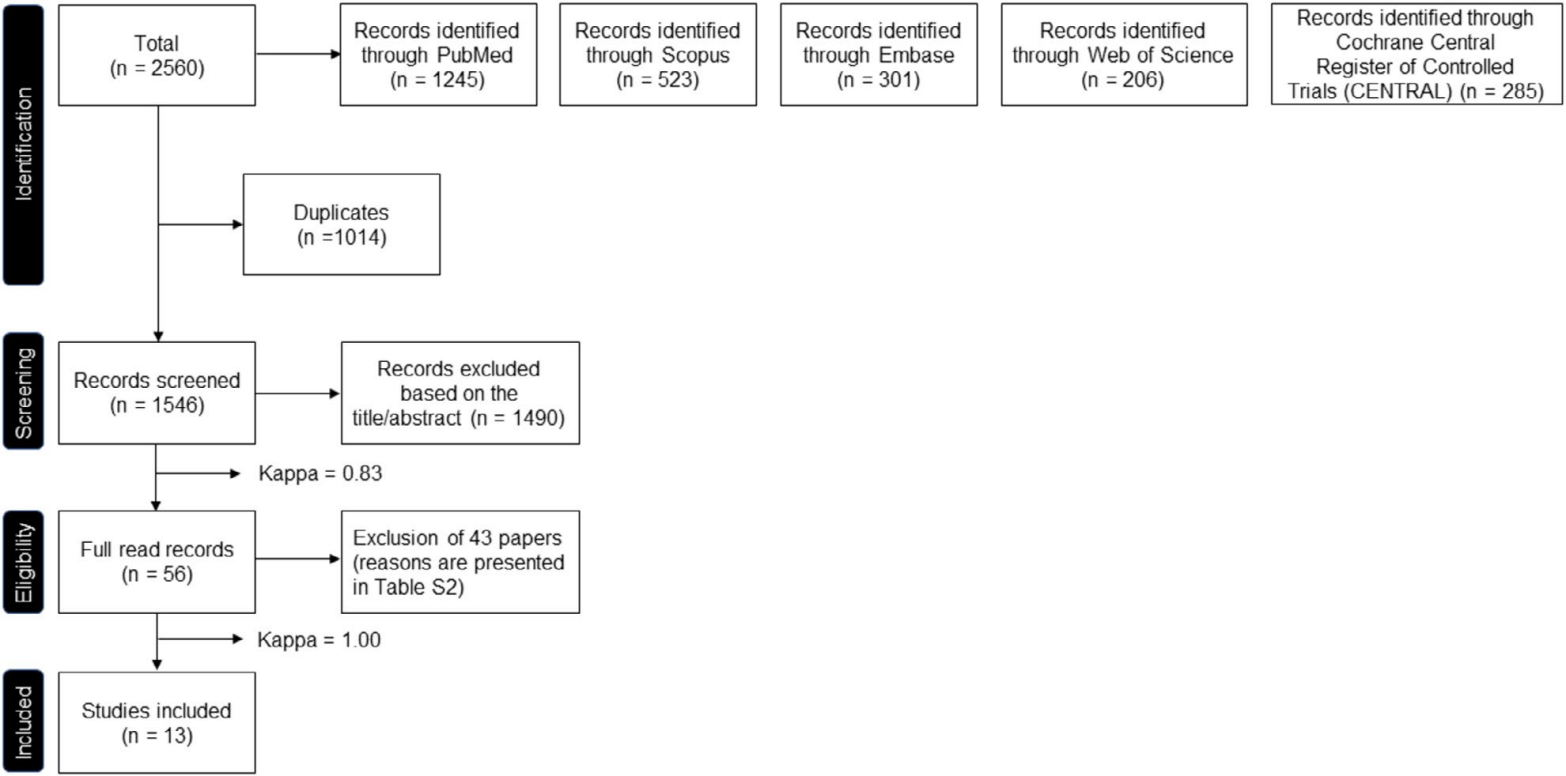
Flowchart with detailed selection process.

The flowchart of the entire selection process is reported in Figure 1. The literature search resulted in 2560 titles. After the exclusion of duplicates, 1014 titles were screened, out of which 56 potentially relevant articles were selected. Following full‐text reading, 43 articles were excluded, and 13 fulfilled the eligibility criteria and were used for data extraction. Inter‐rater reliability, quantified by the Kappa statistic, was 0.83 for the title and abstract screening stage and 1.00 for the full‐text review. The reasons for exclusion are presented in Table S2.

### 3.2 General Characteristics of the Included Studies

Out of the 13 included studies, 1 was an RCT (Menini et al. 2016), and 12 were case series (Yamada et al. 2015; Misumi et al. 2015; Fürhauser et al. 2016; Zhang et al. 2016; Erkapers et al. 2017; Boven et al. 2017; Zembic et al. 2019; Fonteyne et al. 2019; Van Doorne et al. 2020; Testori et al. 2021; Pomares‐Puig et al. 2023; Bouhy et al. 2023). The studies were performed in Belgium (Fonteyne et al. 2019; Van Doorne et al. 2020; Bouhy et al. 2023), the Netherlands (Boven et al. 2017; Zembic et al. 2019), the United States and Sweden (Erkapers et al. 2017), Austria (Fürhauser et al. 2016), Italy (Menini et al. 2016; Testori et al. 2021), Japan (Yamada et al. 2015; Misumi et al. 2015), Spain (Pomares‐Puig et al. 2023), and China (Zhang et al. 2016). The study's maximum follow‐up ranged from 48 h after implant placement (Menini et al. 2016) to 10 years (Zhang et al. 2016; Table 1).

**TABLE 1:** Characteristics of the included studies.

Study	Country	Study setting (university or private practice)	Study design	Number of arms	Funding	Number of patients (included/baseline/last follow‐up)	Sex (female/male)	Mean age (years)	Study's maximum follow‐up	Primary outcome
Bouhy et al. (2023)	Belgium	University	Case series	One	Yes	30/29/27	13/17	66.4 ± 7.7 years (range: 48–82 years)	5 years after implant placement	Prosthodontic survival rate
Boven et al. (2017)	Netherlands	University	Case series	One	Yes	50/50/45	26/24	58.75 ± 9.03 years (range: 42–74 years)	5 years after implant placement	NR
Erkapers et al. (2017)	United States and Sweden	University	Case series	One	Yes	51/51/45	27/24	65.8 (range: 47–83 years)	3 years after implant placement	OHIP‐49 scores
Fonteyne et al. (2019)	Belgium	University and private practice	Case series	One	No	32/30/25	13/17	62.2 ± 9.0 years	6 months after implant placement (on the day the final prosthesis was installed)	NR
Fürhauser et al. (2016)	Austria	University	Case series	One	No	50	27/23	59.75 years	1 week after implant placement	NR
Menini et al. (2016)	Italy	University	RCT	Two (Split‐mouth RCT)	No	11	8/3	63.4 years (range: 51–75 years)	48 h after implant placement	Swelling and pain control
Misumi et al. (2015)	Japan	University	Case series	One	No	10	3/7	62 years (range: 53–76 years)	3 months after the definitive prosthesis placement	OHIP‐14 scores
Pomares‐Puig et al. (2023)	Spain	Private practice	Case series	One	No	10	3/7	56.6 ± 11.02 years	7 days after implant placement	Accuracy of implant placement
Testori et al. (2021)	Italy	Private practice	Case series	One	No	10	6/4	71.10 ± 11.02 years (range: 59–87 years)	1 year^a^	NR
Van Doorne et al. (2020)	Belgium	University	Case series	One	Yes	31/31/29	14/17	62.30 ± 9.28 years	2 years^a^	NR
Yamada et al. (2015)	Japan	Private practice	Case series	One	Yes	50/48/48	22/26	56.0 ± 8.3 years (range: 34–74 years)	1 year^a^	NR
Zembic et al. (2019)	Netherlands	University	Case series	One	Yes	21/21/16	6/15	63 years (range: 52–81 years)	4 years	NR
Zhang et al. (2016)	China	University	Case series	One	Yes	12/11	8/4	56.3 years (range: 40–73 years)	10 years	NR

^a^ PROMs and/or CROMs were not evaluated at the study's maximum follow‐up period.

The number of included patients ranged from 10 (Testori et al. 2021; Pomares‐Puig et al. 2023) to 51 (Erkapers et al. 2017). The patients' mean age ranged from 56 (Yamada et al. 2015) to 71.10 years (Testori et al. 2021). The primary outcome was reported in 5 (Misumi et al. 2015; Menini et al. 2016; Erkapers et al. 2017; Pomares‐Puig et al. 2023; Bouhy et al. 2023) out of 13 studies. PROMs were the primary outcomes in two studies (Misumi et al. 2015; Erkapers et al. 2017; Table 1).

The number of implants ranged from 42 (Zembic et al. 2019) to 306 (Erkapers et al. 2017). The number of implants per patient ranged from 2 (Zembic et al. 2019) to 8 (Zhang et al. 2016; Pomares‐Puig et al. 2023). The timing of implant placement was delayed in 9 studies (Yamada et al. 2015; Misumi et al. 2015; Zhang et al. 2016; Erkapers et al. 2017; Boven et al. 2017; Zembic et al. 2019; Fonteyne et al. 2019; Van Doorne et al. 2020; Bouhy et al. 2023), immediate in two studies (Fürhauser et al. 2016; Pomares‐Puig et al. 2023), and two studies performed both protocols, delayed and immediate implants (Menini et al. 2016; Pomares‐Puig et al. 2023). In eight studies, the implants were placed only in the maxilla (Yamada et al. 2015; Zhang et al. 2016; Erkapers et al. 2017; Boven et al. 2017; Zembic et al. 2019; Fonteyne et al. 2019; Van Doorne et al. 2020; Bouhy et al. 2023); in one study, the implants were installed in the maxilla or maxilla and mandible (Misumi et al. 2015), and in three studies, the implants were installed in the maxilla and mandible (Menini et al. 2016; Testori et al. 2021; Pomares‐Puig et al. 2023; Table 2).

**TABLE 2:** Characteristics of the implants and prostheses.

Study	Timing of implant placement	Timing of loading	Bone grafting	Implants placed only in maxilla or maxilla and mandible	Number of implants (baseline/last follow‐up)	Number of implants per patient	Implant type	Type of prosthesis (fixed or removable)
Bouhy et al. (2023)	Delayed	Delayed loading	No	Maxilla	116/108	4	Standard implants	Removable (overdenture)
Boven et al. (2017)	Delayed	Delayed loading	In some cases, a sinus lifting procedure was performed using autogenous bone prior to implant placement, and implants were placed after the healing period	Maxilla	300/295	6	Standard implants	Removable (overdenture)
Erkapers et al. (2017)	Delayed	Delayed loading	No	Maxilla	306/263	6	Standard implants	Fixed (screw‐retained)
Fonteyne et al. (2019)	Delayed	Delayed loading	No	Maxilla	NR	5–6	Mini‐dental implants	Removable (overdenture)
Fürhauser et al. (2016)	Immediate (implants were placed immediately following extractions, during the same surgical procedure)	Immediate loading	No	Maxilla and mandible	200	4	Standard implants	Fixed (screw‐retained)
Menini et al. (2016)	Some immediate (implants were placed immediately following extractions, during the same surgical procedure) and some delayed	Immediate loading	No	Maxilla and mandible	44	4	Standard implants	Fixed (the method of retention was not reported)
Misumi et al. (2015)	Delayed	Immediate loading	No	Maxilla or maxilla and mandible	65	4–6	Standard implants	Fixed (screw‐retained)
Pomares‐puig et al. (2023)	Some immediate (implants were placed immediately following extractions, during the same surgical procedure) and some delayed	Immediate loading	In some cases, using a mixture of autogenous bone, xenograft and a collagen membrane	Maxilla and mandible	48	4–8^a^	Standard implants	Fixed (screw‐retained)
Testori et al. (2021)	Immediate (implants were placed immediately following extractions, during the same surgical procedure)	Immediate loading	In some cases, using xenograft and/or collagen membrane or L‐PRF	Maxilla and mandible	46/45	4–7	Standard implants	Fixed (screw‐retained)
Van Doorne et al. (2020)	Delayed	Delayed loading	No	Maxilla	185/166	5–6	Mini‐dental implants	Removable (overdenture)
Yamada et al. (2015)	Delayed	Immediate loading	No	Maxilla	290/278	4–6	Standard implants	Fixed (screw‐retained)
Zembic et al. (2019)	Delayed	Delayed loading	In some cases. In case of minor bone defects, guided bone regeneration procedures not compromising primary implant stability were applied. The materials used were not reported	Maxilla	42/32	2	Standard implants	Removable (overdenture)
Zhang et al. (2016)	Delayed	Early loading	No	Maxilla	91/83	6–8	Standard implants	Fixed (segmented, cement‐retained)

^a^ Some patients received implants and their corresponding implant‐supported restorations in both the maxilla and mandible.

Eleven studies used standard implants (Yamada et al. 2015; Misumi et al. 2015; Fürhauser et al. 2016; Menini et al. 2016; Zhang et al. 2016; Erkapers et al. 2017; Boven et al. 2017; Zembic et al. 2019; Testori et al. 2021; Pomares‐Puig et al. 2023; Bouhy et al. 2023), and two used mini‐dental implants (Fonteyne et al. 2019; Van Doorne et al. 2020). Five studies used removable prostheses (Boven et al. 2017; Zembic et al. 2019; Fonteyne et al. 2019; Van Doorne et al. 2020; Bouhy et al. 2023), and eight studies used fixed prostheses (Yamada et al. 2015; Misumi et al. 2015; Fürhauser et al. 2016; Menini et al. 2016; Zhang et al. 2016; Erkapers et al. 2017; Testori et al. 2021; Pomares‐Puig et al. 2023; Table 2).

Due to the variety of outcomes and measurements, results were organized into PROs/PROMs and ClinROs/CROMs, detailing the outcomes and their respective assessment methods.

### 3.3 Risk of Bias

**FIGURE 2:**
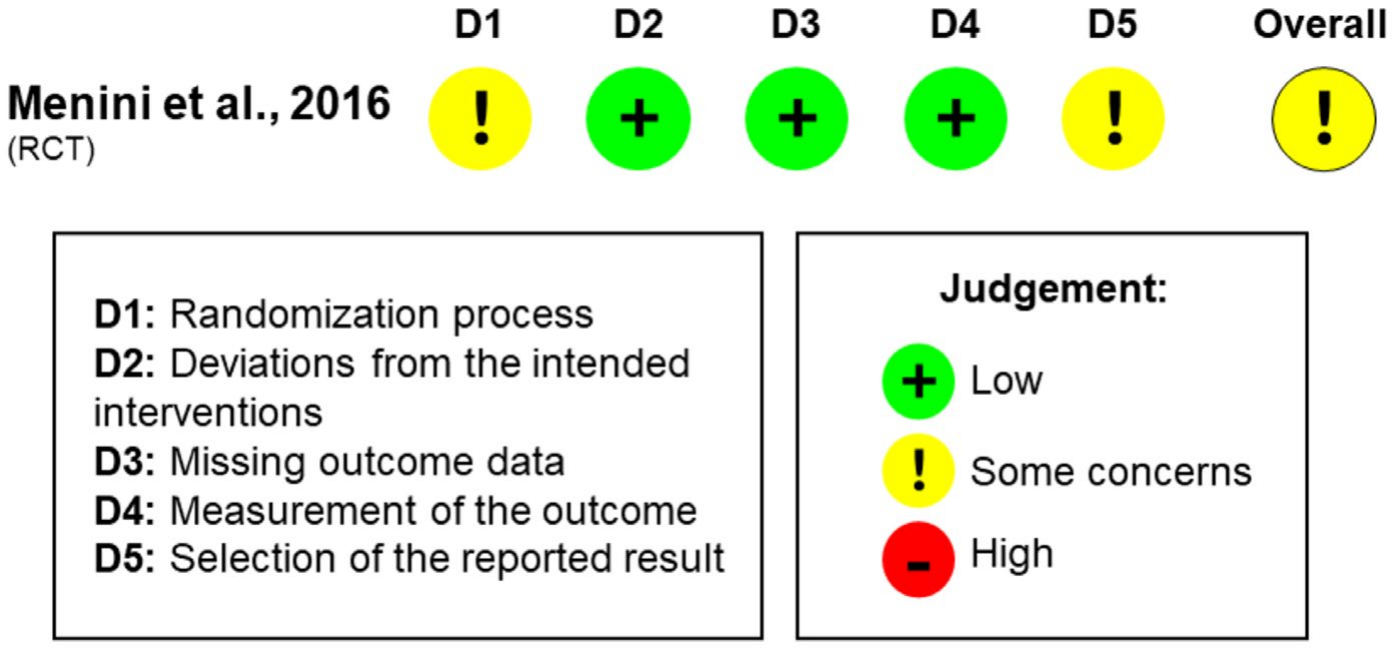
Risk of bias assessment of the included RCT.

The randomized controlled trial was categorized as having some concerns according to the RoB2 tool (Menini et al. 2016). These concerns stemmed from the lack of information regarding allocation sequence concealment (Domain 1) and the absence of a registered protocol (Domain 5; Figure 2).

**FIGURE 3:**
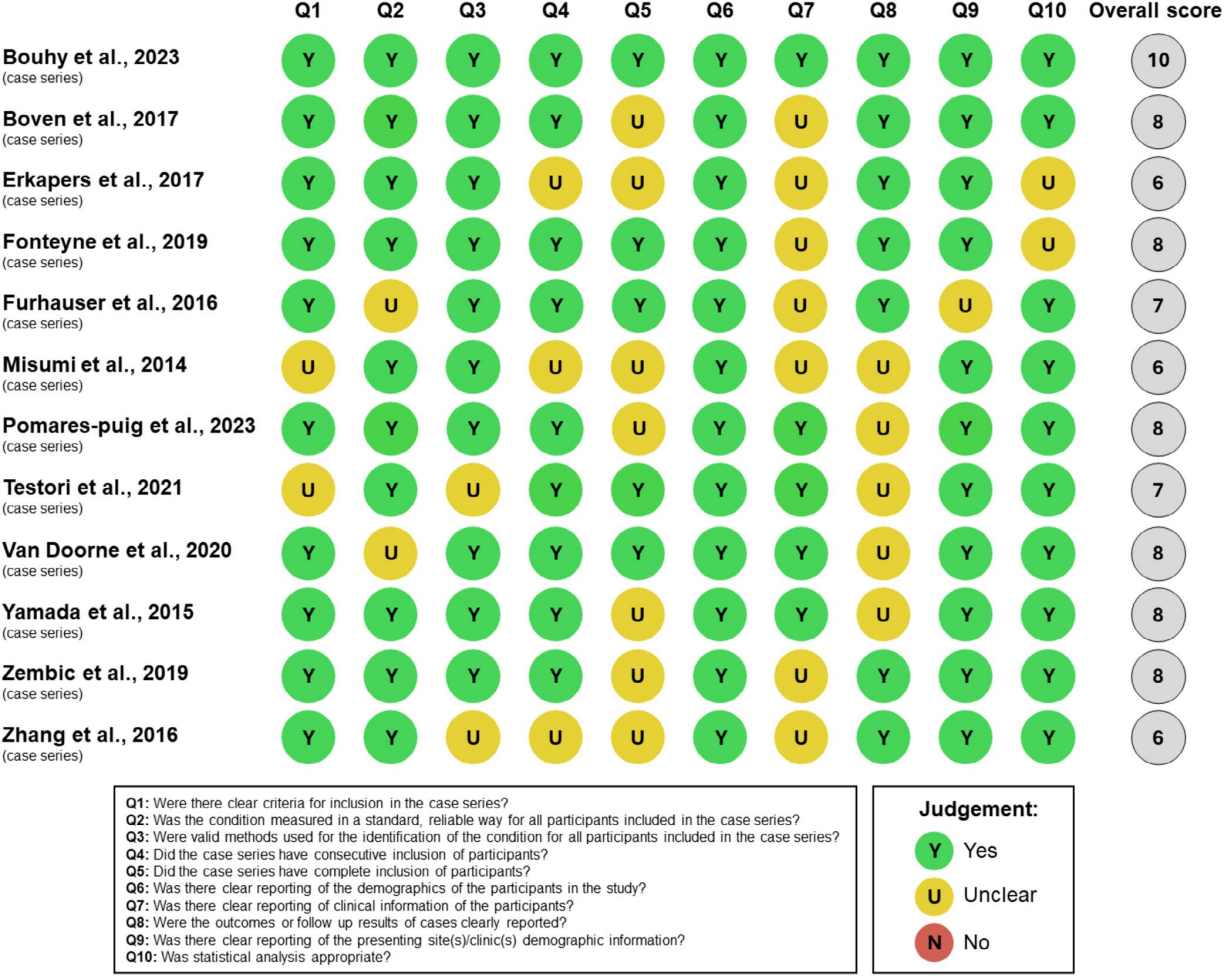
Methodological quality assessment of the included case series.

For the 12‐case series, quality assessment using the JBI 10‐point checklist revealed a mean quality score of 7.5. One case series received the highest score (10/10) (Bouhy et al. 2023), 6 received 8/10 (Yamada et al. 2015; Boven et al. 2017; Zembic et al. 2019; Fonteyne et al. 2019; Van Doorne et al. 2020; Pomares‐Puig et al. 2023), 2 received 7/10 (Fürhauser et al. 2016; Testori et al. 2021), and 3 received 6/10 (Misumi et al. 2015; Zhang et al. 2016; Erkapers et al. 2017). The highest‐scoring criteria were clearly describing the patient's demographic characteristics (12/12) and site/clinic demographic information (11/12). The lowest scores were for complete inclusion of participants (7/12), clear reporting of outcomes or follow‐up results (7/12), and clear reporting of clinical information of the participants (5/12; Figure 3).

### 3.4 PROs and PROMs

The following domains or key aspects were analyzed using respective methods, ordered by frequency of assessment:

1. *Oral health‐related quality of life*, encompassing the domains of functional limitation, physical pain, psychological discomfort, physical disability, psychological disability, social disability, and handicap, was evaluated in 7 out of 13 studies, accounting for 53.85% of the total studies reviewed. This assessment used various versions of the Oral Health Impact Profile (OHIP):
  - OHIP‐14 (14‐item questionnaire) and its variant OHIP‐J14 were applied in 3 studies, constituting 23.08% (Misumi et al. 2015; Fonteyne et al. 2019; Pomares‐Puig et al. 2023).
  - OHIP‐20 or OHIP‐EDENT (20‐item questionnaire) was employed in 2 studies, representing 15.38% of the total (Zembic et al. 2019; Bouhy et al. 2023).
  - OHIP‐49 (49‐item questionnaire) was used in 1 study, accounting for 7.69% (Erkapers et al. 2017).
  - The OHIP‐J49 (54‐item questionnaire, which includes additional Japanese‐specific questions) was used in 1 study, representing 7.69% (Yamada et al. 2015).
2. *Pain intensity* was analyzed in 5 out of 13 studies, accounting for 38.46% of the total studies reviewed. This was assessed using:
  - Postoperative pain, using a 0–100 mm VAS in 2 studies, representing 15.38% (Yamada et al. 2015; Pomares‐Puig et al. 2023).
  - Postoperative pain, using a 10 cm VAS in 1 study, representing 7.69% (Fürhauser et al. 2016).
  - Postoperative pain, using a verbal rating scale (VRS) with six levels (categories) in 1 study, represents 7.69% (Menini et al. 2016).
  - Postoperative pain, using a numeric rating scale (NRS) from 1 to 10 and after, classified according to scores in 1 study, representing 7.69% (Van Doorne et al. 2020).
  - Intraoperative pain, using a 0–100 mm VAS in 1 study, represents 7.69% (Pomares‐Puig et al. 2023).
3. *Patient satisfaction with the prosthesis* was analyzed in 3 out of 13 studies, accounting for 23.08% of the total studies reviewed. This was assessed using
  - A 100 mm visual analog scale (VAS) in 2 studies, representing 15.38% of the total (Zembic et al. 2019; Bouhy et al. 2023).
  - A questionnaire with multiple‐choice options (categories) in 1 study, representing 7.69% of the total (Zhang et al. 2016).
4. *Postoperative drug administration* was analyzed in 2 out of 13 studies, representing 15.38%, by recording postoperative drug use (Menini et al. 2016; Pomares‐Puig et al. 2023).
5. *Patient overall satisfaction with the treatment* was analyzed in 1 study (7.69%), using a 10‐point rating scale (Boven et al. 2017).
6. *Overall satisfaction with oral health* was analyzed in 1 study (7.69%), using a 10 cm VAS (Fonteyne et al. 2019).
7. *Patient well‐being during the surgical procedure* was assessed in 1 study (7.69%), using a questionnaire with a Likert scale ranging from −2 to 2 (Pomares‐Puig et al. 2023).
8. *Satisfaction during the surgery* was assessed in 1 study (7.69%), using a 0–100 mm VAS (Pomares‐Puig et al. 2023).
9. *Psychosocial impact (comfort) using a device dedicated to managing postoperative swelling and pain* was analyzed in 1 study (7.69%), using a numerical rating scale (NRS) from 0 to 10 (Menini et al. 2016).
10. *Postoperative impairment of everyday life and work* was analyzed in 1 study (7.69%) using a 10 cm VAS (Fürhauser et al. 2016).
11. *Postsurgical quality of life* was assessed in 1 study (7.69%), using a questionnaire with multiple‐choice options, scored from 0 to 10 (Testori et al. 2021).
12. *Postprosthetic quality of life* was assessed in 1 study (7.69%), using a questionnaire with multiple‐choice options, scored from 0 to 10 (Testori et al. 2021).
13. *Functional complaints about the prosthesis* were analysed in 1 study (7.69%), using a 7‐item questionnaire with a four‐point rating scale with multiple‐choice options (scores; Boven et al. 2017).
14. *Patient's eating ability* was analyzed in 1 study (7.69%), using a questionnaire with multiple‐choice options (scores; Boven et al. 2017).
15. *Speech measured by one OHIP‐14 question* from the domain “functional limitation” was analysed in 1 study (7.69%; Fonteyne et al. 2019).
16. *Satisfaction with speech* was analyzed in 1 study (7.69%), using a 10 cm VAS (Fonteyne et al. 2019).

PROs related to the immediate postoperative period (up to 1 week) were consistently evaluated in studies with immediate implant placement. However, similar outcomes were also reported in studies with delayed implant placement.

Further details on the PROs and PROMs are provided in Table 3, while references for the methods cited in the respective studies, along with information on validation, can be found in Table S3.

**TABLE 3:** Patient‐reported outcomes.

Study	Timing of implant placement	Patient‐reported outcome measures (PROMs)	Evaluated question (key aspects/domains)	Methods	Interpretation	Analysis metric	Method of aggregation	Time point	Pre‐/Post‐intervention analysis (PROs/PROMs)
Bouhy et al. (2023)	Delayed	Oral Health Impact Profile 20 (OHIP‐20) Patient satisfaction questionnaire—An adaptation of the McGill Denture Satisfaction	Oral health‐related quality of life (domains: functional limitation, physical pain, psychological discomfort, physical disability, psychological disability, social disability, and handicap) Patient satisfaction with the prosthesis	**OHIP‐20 (OHIP‐EDENT):** A 20‐item questionnaire that measures self‐reported patients' oral health‐related quality of life. The items were rated on six‐point Likert scales (“never” = 1, “rarely” = 2, “occasionally” = 3, “often” = 4, “very often” = 5, “all of the time” = 6) **Patient satisfaction:** An adaptation of the McGill Denture Satisfaction scale, using a 100 mm VAS illustrated by “not at all satisfied” on the left side and “extremely satisfied” on the right side. Six aspects were assessed: general comfort, stability, ability to chew, speech, cleaning ability, and pain	**OHIP‐20:** The total range is 20–120 points, with lower scores indicating better oral health‐related quality of life, and higher scores indicating more significant impacts on the patient's daily life due to oral health issues **Patient satisfaction:** The left end (0 mm) represents “not at all satisfied,” while the right end (100 mm) represents “extremely satisfied”	Point estimates at each time point	**OHIP‐20 scores (domains and overall):** Reported as mean (SD) **VAS scores:** Reported as mean (SD)	Baseline (before implant placement), 1, 3, and 5 years after implant placement	**OHIP‐20 scores:** Pre−/post‐intervention analysis **VAS scores:** Pre−/post‐intervention analysis
Boven et al. (2017)	Delayed	Functional complaints about the prosthesis Patients' eating ability Patient overall satisfaction with the treatment	Functional complaints about the prosthesis (**f**unctional complaints in general, facial aesthetics, ‘neutral space’, aesthetics, soft food, tough food, hard food) Patients' eating ability (how well the patient could eat soft, tough, and hard food) Patient overall satisfaction with the treatment	**Functional complaints about the upper denture:** A 7‐item questionnaire. The extent of each specific complaint can be expressed on a four‐point rating scale (0 = “no complaints”; 1 = “little complaints”; 2 = “moderate complaints”; and 3 = “severe complaints”) **Patients' eating ability with their upper denture:** Assessed using a chewing ability questionnaire. This questionnaire focused on how well the patient could eat soft, tough, and hard food. There were three possible answers (0 = “good”; 1 = “moderate”; and 2 = “bad”) **Patients' overall satisfaction:** 10‐point rating scale (1 = “very bad” to 10 = “excellent”)	**Functional complaints about the upper denture:** The higher the score, the more severe the complaint **Patients' eating ability:** The higher the score, the greater the difficulties the patient experiences with eating **Patient overall satisfaction:** The higher the score, the greater the patient's overall satisfaction with the treatment	Point estimates at each time point	**Functional complaints:** Reported as median (IQR) **Eating ability:** Reported as median (IQR) **Patient overall satisfaction (score):** Reported as mean (SD)	Baseline (before implant placement), and 5 years after implant placement	**Functional complaints:** Pre−/post‐intervention analysis **Eating ability:** Pre−/post‐intervention analysis **Patient overall satisfaction (score):** Pre−/post‐intervention analysis
Erkapers et al. (2017)	Delayed	Oral Health Impact Profile (OHIP‐49)	Oral health‐related quality of life (domains: functional limitation, physical pain, psychological discomfort, physical disability, psychological disability, social disability, and handicap)	**OHIP‐49:** A 49‐item questionnaire that measures self‐reported patients' oral health‐related quality of life. Each question offers five response categories: “never” = 0, “hardly ever” = 1, “sometimes” = 2, “fairly often” = 3, and “very often” = 4	**OHIP‐49:** The total range is 0–196 points, with lower scores indicating better oral health‐related quality of life, and higher scores indicating more significant impacts on the patient's daily life due to oral health issues	Point estimates at each time point	**OHIP‐49 (domains and overall score):** Reported as mean (SD)	Baseline (before implant placement), 12 weeks, 6 months, 1, 2 and 3 years after implant placement	**OHIP‐49** (domains and overall score) Pre−/post‐intervention analysis
Fonteyne et al. (2019)	Delayed	Oral Health Impact Profile (OHIP‐14) Speech measured by one OHIP‐14 question Overall satisfaction with oral health Satisfaction with speech	Phonetic evaluation (articulation and oromyofunctional behavior) Oral health‐related quality of life (domains: functional limitation, physical pain, psychological discomfort, physical disability, psychological disability, social disability, and handicap) Overall satisfaction with oral health Satisfaction with speech	**OHIP‐14 (Dutch version):** A 14‐item questionnaire that measures self‐reported patients' oral health‐related quality of life. Each question offers five response categories: “never” = 0, “hardly ever” = 1, “sometimes” = 2, “fairly often” = 3, and “very often” = 4 **Speech (measured by one OHIP‐14 question):** One question of the domain ‘functional limitation’ (Have you had trouble pronouncing any words because of problems with your teeth, mouth, dentures or jaw?) was used to determine the impact of the prosthesis on speech. The items were rated by a Likert scale ranging from 0 (no discomfort) to 4 (high discomfort) **Overall satisfaction with oral health:** 10 cm VAS, with one end reflecting 100% maximal satisfaction and the other end corresponding to 0% complete dissatisfaction **Satisfaction with speech:** 10 cm VAS, with one end reflecting 100% maximal satisfaction and the other end corresponding to 0% complete dissatisfaction	**OHIP‐14:** The total range is 0–56. A score of 56/56 is indicative for maximal negative appreciation and 0/56 indicates that there are no issues at all **Overall satisfaction with oral health:** One end (10 cm) represents 100% maximal satisfaction with oral health, while the other end (0 cm) represents 0% complete dissatisfaction **Satisfaction with speech:** One end (10 cm) represents 100% satisfaction with speech and 0 cm represents complete dissatisfaction	Point estimates at each time point	**OHIP‐14 (overall score):** reported as mean (SD) and mean differences **Speech measured by one OHIP question:** Frequency of “never”, “hardly ever”, “occasionally”, “fairly often” and “very often” **Overall satisfaction with oral health (VAS score):** reported as mean (SD) and mean differences **Satisfaction with speech (VAS score):** reported as mean (SD) and mean differences	Beseline (before implant placement), provisional loading, final connection (6 months after implant placement)	**OHIP‐14 (overall score):** Pre−/post‐intervention analysis **Speech measured by one OHIP question:** Pre−/post‐intervention analysis **Overall satisfaction with oral health (VAS score):** Pre−/post‐intervention analysis **Satisfaction with speech (VAS score):** Pre−/post‐intervention analysis
Fürhauser et al. (2016)	Immediate	Postoperative pain intensity Postoperative swelling Impairment in everyday life and work after surgery	Postoperative pain intensity Postoperative swelling Impairment in everyday life and work after surgery (days until resuming everyday life and work, perception of temporary prosthesis as a foreign body, impairment regarding food intake after surgery, impairment due to prosthetic complications, impairment regarding speech after surgery, and willingness to undergo the procedure again)	**Postoperative pain intensity and swelling:** VAS from 0 (none) to 10 (severe) **Postoperative impairment of everyday life and work:** VAS from 0 to 10	**Pain intensity and swelling:** Categorized as “no pain/swelling” (score 0), “minor pain/swelling” (scores 1–5), and “major pain/swelling” (scores 6–10) **Postoperative impairment of everyday life and work:** Higher scores generally indicate more significant impairments or stronger perceptions, with 0 representing no impairment (or not at all) and 10 representing massive impairment (or anytime for willingness). Except in the case of willingness to undergo the procedure again, where higher scores indicate greater satisfaction	Point estimates at each time point	**Pain intensity and swelling:** Reported as the percentage of patients experiencing no pain/swelling (score 0), minor pain/swelling (scores 1–5), and major pain/swelling (scores 6–10) **Impairment in everyday life and work after surgery:** Reported as mean (SD) score for each question, along with the observed minimum and maximum scores	**Pain intensity and swelling:** Assessed on the day of the surgery, and on the first and second days after surgery **Impairment in everyday life and work after surgery:** Assessed 1 week after surgery	**Pain intensity and swelling:** Post‐intervention analysis **Impairment in everyday life and work after surgery:** Post‐intervention analysis
Menini et al. (2016)	Some immediate and some delayed	Pain Psychosocial impact (comfort) of using the device RecoveryRx Postoperative drug use.	Pain Psychosocial impact (comfort) of using the RecoveryRx device, a non‐invasive, disposable patch for managing postoperative swelling and pain with a 27.12 MHz electromagnetic field, was evaluated based on invasiveness, hygiene maneuvers, alimentation, and rest Postoperative drug use (type of non‐steroidal anti‐inflammatory drug (NSAIDs) and doses taken)	**Pain:** Verbal rating scale (VRS) with six levels: no pain, slight pain, moderate pain, severe pain, very severe pain, and the worst possible pain **Psychosocial impact (comfort) of using the device RecoveryRx:** 11‐point numerical rating scale (NRS), with 0 indicating no discomfort and 10 indicating maximum discomfort. Patients rated their discomfort for each item **Postoperative drug administration:** Data on postoperative drug administration was recorded	**Pain:** Classified into six levels: no pain, slight pain, moderate pain, severe pain, very severe pain, and the worst possible pain **Psychosocial impact:** The higher the number, the higher the discomfort **Postoperative drug administration:** A higher dose of drug suggests more pain	Point estimates at each time point	**Pain:** Frequency of occurrences in each category: none, slight, moderate, severe, very severe, and worst pain **Psychosocial Impact (Comfort):** Percentage distribution of scores on the NRS scale **Postoperative Drug Administration:** Description of the drug, dose, and time of consumption	**Pain:** Assessed 48 h after the surgery **Psychosocial impact:** Assessed 48 h after the surgery **Postoperative drug administration:** Assessed 0–24 and 24–48 h after the surgery	**Pain:** Post‐intervention analysis **Psychosocial impact:** Post‐intervention analysis **Postoperative drug administration:** Post‐intervention analysis
Misumi et al. (2015)	Delayed	Oral Health Impact Profile (OHIP‐J14)	Oral health‐related quality of life (domains: functional limitation, physical pain, psychological discomfort, physical disability, psychological disability, social disability, and handicap)	**OHIP‐14 (Japanese version):** A 14‐item questionnaire that measures self‐reported patients' oral health‐related quality of life. Each question offers five response categories: “never” = 0, “hardly ever” = 1, “sometimes” = 2, “fairly often” = 3, and “very often” = 4	The total range is 0–56. A score of 56/56 is indicative for maximal negative appreciation and 0/56 indicates that there are no issues at all	Point estimates at each time point	**OHIP‐14 (domains and overall score):** Reported as mean (SD)	Baseline (before implant placement), 1 week after immediate restoration placement, 1 week after secondary provisional restoration placement, and 3 months after final restoration placement	**OHIP‐14 (domains and overall score):** Pre−/post‐intervention analysis
Pomares‐puig et al. (2023)	Some immediate and some delayed	Patient well‐being during the surgical procedure Intraoperative pain Satisfaction during the surgery Oral Health Impact Profile (OHIP‐14) Intake of analgesic and anti‐inflammatory medication Postoperative pain	Patient well‐being during the surgical procedure (questions were related to the duration of the surgery, discomfort, ease of keeping the mouth open, willingness to repeat the procedure, recommendation of the procedure to others, and perceived accuracy and results of the guided technique) Oral health‐related quality of life (domains: functional limitation, physical pain, psychological discomfort, physical disability, psychological disability, social disability, and handicap) Intraoperative pain Satisfaction during the surgical procedure Intake of analgesics and anti‐inflammatory medication and postoperative pain	**Patient well‐being during the surgical procedure:** A questionnaire that assessed the duration of surgery; discomfort from liquids, instruments, and devices in the mouth; ease of keeping the mouth open, willingness to repeat the procedure; discomfort from vibrations; likelihood of recommending the procedure, and perceived accuracy and results with computer‐guided surgery. Responses were given on a Likert scale ranging from “absolutely disagree” to “absolutely agree”, with scores from −2 to 2 based on the positivity of the response **Intraoperative pain:** 0‐100 mm VAS, where 0 mm indicates no pain and 100 mm indicates the worst possible pain **Satisfaction during the surgery:** 0–100 mm VAS, where 0 mm indicates complete dissatisfaction and 100 mm indicates complete satisfaction **OHIP‐14 (Spanish version):** A 14‐item questionnaire that measures self‐reported patients' oral health‐related quality of life. Each question offers five response categories: “never” = 0, “hardly ever” = 1, “sometimes” = 2, “fairly often” = 3, and “very often” = 4 **Intake of analgesic and anti‐inflammatory medication:** Number of analgesic medication intake for 1 week **Postoperative pain:** 0–100 mm VAS, where 0 mm indicates no pain and 100 mm indicates the worst possible pain	**Patient well‐being during the surgical procedure:** Positive scores indicate favorable experiences, while negative scores highlight discomfort and dissatisfaction **Intraoperative pain scores:** Lower scores suggest minimal pain, while higher scores indicate greater pain experienced during the procedure **Satisfaction during the surgery:** Lower scores indicate dissatisfaction, while higher scores indicate greater satisfaction **OHIP‐14 (only the overall score):** Lower scores reflect better oral health‐related quality of life, while higher scores indicate poorer quality of life **Intake of analgesic and anti‐inflammatory medication:** A higher number of medications suggests more pain **Postoperative pain:** Lower scores suggest minimal pain, while higher scores indicate greater pain experienced during the procedure	Point estimates at each time point	**Patient well‐being during the surgical procedure:** Reported as frequency of positive, neutral or negative rating **Intraoperative pain scores:** Reported as mean (SD) **Satisfaction during the surgery:** Reported as mean (SD) **OHIP‐14 (overall score):** Reported as median (IQR) and mean differences between the time points **Intake of analgesic and anti‐inflammatory medication:** Reported as median (IQR) **Postoperative pain:** Reported as median (IQR)	**Patient well‐being during the surgical procedure:** Assessed immediately after the surgery **Intraoperative pain:** Assessed immediately after the surgery **Satisfaction during the surgery:** Assessed before implant placement and immediately after surgery **OHIP‐14:** Assessed 1 week after the surgery (at Day 0) **Intake of analgesic and anti‐inflammatory medication:** Assessed 1 week after surgery (Day 0–7) **Postoperative pain:** Assessed 1 week after surgery (Day 0–7)	**Patient well‐being during the surgical procedure:** Post‐intervention analysis **Intraoperative pain scores:** Post‐intervention analysis **Satisfaction during the surgery:** Pre−/post‐intervention analysis **OHIP‐14 (overall score):** Post‐intervention analysis **Intake of analgesic and anti‐inflammatory medication:** Post‐intervention analysis **Postoperative pain:** Post‐intervention analysis
Testori et al. (2021)	Immediate	Postsurgery quality of life Postprosthetic quality of life	The postsurgical quality of life questionnaire evaluated pain management (pain and need for painkillers), physical symptoms (swelling, bruising, bleeding), daily functioning (sleep, chewing, speaking), and general well‐being (nausea, taste alterations) The postprosthetic quality of life questionnaire evaluated the overall esthetic result, color, shape, and size of the prosthesis (new teeth), appearance of the gingival tissue, chewing ability, speech, home‐care hygiene performance, and how closely the result met the patient's expectations	**Postsurgical quality of life:** A questionnaire evaluating pain at the treated site, the necessity of taking prescribed painkillers, the presence of swelling or bruising, effects on sleep, alterations in chewing and speaking, bleeding at the treated site, feelings of nausea, and taste alterations. The responses were scored from 0 to 10 **Postprosthetic quality of life:** A questionnaire evaluating the overall esthetic result of the prosthesis; the color, shape, and size of the prosthesis (“new teeth”); the appearance of the gingival tissue; chewing ability, speech, home‐care hygiene performance around the prosthesis, and how closely the result met the patient's expectations. Responses were scored from 0 to 10	**Postsurgery quality of life:** Scores indicating levels of satisfaction: 0–3 for fully satisfied, 4–6 for moderately satisfied, and 7–10 for poorly satisfied **Postprosthetic quality of life:** Scores indicating levels of satisfaction: 0–3 for fully satisfied, 4–6 for moderately satisfied, and 7–10 for poorly satisfied	Point estimates at each time point	**Postsurgery quality of life:** Reported as the frequency of categories fully satisfied, moderately satisfied and poorly satisfied **Postprosthetic quality of life:** Reported as the frequency of categories fully satisfied, moderately satisfied and poorly satisfied	**Postsurgery quality of life:** Assessed 1 week after the implant placement **Postprosthetic quality of life:** Assessed 1 month after the prosthesis placement	**Postsurgery quality of life:** Post‐intervention analysis **Postprosthetic quality of life:** Post‐intervention analysis
Van Doorne et al. (2020)	Delayed	Pain Patient satisfaction	Pain Patient satisfaction during the treatment, assessing inconveniences experienced during the process, and whether the patient would recommend the treatment to others	**Pain:** A numeric rating scale (NRS) from 1 to 10, with 1–3 indicating mild pain, 4–6 indicating moderate pain, and 7–10 indicating severe pain **Patient satisfaction:** A questionnaire about the inconveniences experienced during treatment. Additionally, “whether the patient would recommend the treatment to other patients” was recorded as ‘yes’ or ‘no’ at the final visit	**Pain:** Scores 1–3 indicating mild pain, 4–6 indicating moderate pain, and 7–10 indicating severe pain **Patient satisfaction:** Regarding the recommendation for the treatment, ‘yes’ indicates a positive experience, while a ‘no’ indicates dissatisfaction	Point estimates (scores for pain and categorize satisfaction as ‘yes’ or ‘no’), at each time point	**Pain scores:** Reported as mean (SD) **Patient satisfaction:** Reported as mean (SD) and the recommendation rate reported as the percentage of ‘yes’	**Pain:** The day of implant placement and after 1 week **Patient satisfaction:** Not reported	**Pain:** Post‐intervention analysis **Patient satisfaction:** Not reported
Yamada et al. (2015)	Delayed	Oral Health Impact Profile (OHIP‐J49) Postoperative pain	Oral health‐related quality of life (domains: functional limitation, physical pain, psychological discomfort, physical disability, psychological disability, social disability, and handicap) Postoperative pain	**OHIP‐49 (Japanese version):** A 54‐item questionnaire, including the seven subdomains and additional Japanese items, that measures self‐reported patients' oral health‐related quality of life. Each question offers five response categories: “never” = 0, “hardly ever” = 1, “sometimes” = 2, “fairly often” = 3, and “very often” = 4 **Pain:** 100 mm VAS	**OHIP‐49 (OHIP‐J):** The total score ranges from 0 to 216, with higher scores indicating more severe disability **Pain:** Higher scores on VAS indicate greater postoperative pain severity	Point estimates at each time point	**OHIP‐49 (OHIP‐J – domains and overall score):** Reported as mean (SD) **Pain scores:** Reported as mean (SD)	**OHIP‐49:** Baseline (before implant placement), after insertion of the provisional prosthesis, and after delivery of the definitive prosthesis **Pain:** Not reported	**OHIP‐49 (OHIP‐J – domains and overall score):** Pre−/post‐intervention analysis **Pain scores:** Not reported
Zembic et al. (2019)	Delayed	Oral Health Impact Profile (OHIP‐EDENT) Patient satisfaction with the prosthesis	Oral health‐related quality of life (domains: functional limitation, physical pain, psychological discomfort, physical disability, psychological disability, social disability, and handicap) and the impact of the prosthetic treatment on the quality of life, and patient satisfaction	**OHIP‐EDENT:** A 20‐item questionnaire that measures the oral health‐related quality of life and the impact of prosthetic treatment in edentulous patients. The items were rated on six‐point Likert scales (“never” = 1, “rarely” = 2, “occasionally” = 3, “often” = 4, “very often” = 5, “all of the time” = 6) **Patient satisfaction with the prosthesis:** Using a 100 mm horizontal VAS line, with “never” on the left end representing 100% satisfaction and “always” on the right end representing 0% satisfaction. Each patient marked their satisfaction by placing a vertical stripe on the line, which was then measured in millimeters	**OHIP‐EDENT:** Lower OHIP‐20 score suggests that the patient has a better perception of their oral health and its impact on their quality of life, whereas a higher score indicates more severe problems and a greater negative impact on their quality of life **Patient satisfaction with the prosthesis:** Higher millimeter values indicated reduced patient satisfaction	Point estimates at each time point	**OHIP‐EDENT (domains and overall score):** Reported as mean (SD) **Patient satisfaction (scores):** Reported as mean (SD)	**OHIP‐EDENT:** Assessed at 2 months, 1 and 4 years **Patient satisfaction:** Baseline (with the old denture), at the delivery of the new denture, 2 months after the delivery of the new denture	**OHIP‐EDENT:** Post‐intervention analysis **Patient satisfaction:** Pre−/post‐intervention analysis
Zhang et al. (2016)	Delayed	Patient satisfaction with the prosthesis	Patient satisfaction, **d**etermined by asking them verbal questions about the appearance, comfort, ability to masticate, and general satisfaction	**Patient satisfaction:** Patients were asked to grade those parameters as excellent, good, fair, or poor	Ratings of “excellent” and “good” reflect positive satisfaction, while “fair” and “poor” indicate areas where the patient experienced dissatisfaction or challenges.	Point estimates at each time point	**Patient satisfaction:** Frequency of the categories	Baseline (immediately after prosthesis placement), 1, 3, 5, and 10 years.	**Patient satisfaction:** Pre−/post‐intervention analysis

### 3.5 ClinROs and CROMs

Ten of the included studies assessed ClinROs. The following domains or key aspects were evaluated using respective methods, listed in order of their frequency of assessment:

1. *Implant survival* was reported in five studies (27.8%), defined as the clinical presence of the implant without signs of mobility (Boven et al. 2017; Van Doorne et al. 2020; Testori et al. 2021; Bouhy et al. 2023) or, in addition to the aforementioned criteria, the absence of discomfort, symptoms of neurologic problems, or signs of peri‐implantitis (Yamada et al. 2015).
2. *Prosthetic complications* were reported in four studies (22.2%), including abutment loosening, prosthesis misfit, veneer chipping, fractures, screw loosening, significant wear, loss, or wear of the matrix component (in overdentures), need for prosthesis modification, and others (Yamada et al. 2015; Zhang et al. 2016; Erkapers et al. 2017; Bouhy et al. 2023).
3. *Prosthetic survival* was reported in three studies (16.7%), defined as a prosthesis in function without the need for replacement (Yamada et al. 2015; Van Doorne et al. 2020; Testori et al. 2021).
4. *Peri‐implant bone level changes—marginal bone loss* was reported in three studies (16.7%), assessed using standardized intraoral radiographs (Yamada et al. 2015; Zhang et al. 2016) and panoramic radiographs (Boven et al. 2017).
5. *Probing depth* was reported in three studies (16.7%), measured at multiple sites per implant using calibrated periodontal probes (Zhang et al. 2016; Boven et al. 2017; Bouhy et al. 2023).
6. *Modified plaque index or plaque index* was reported in three studies, scored from 0 to 3 according to established criteria (16.7%) (Zhang et al. 2016; Boven et al. 2017; Bouhy et al. 2023).
7. Sulcular Modified Bleeding Index was reported in three studies (16.7%), scored from 0 to 3 according to established criteria (Zhang et al. 2016; Boven et al. 2017; Bouhy et al. 2023).
8. *Swelling* was analyzed in two studies (11.1%), using standardized frontal and side images, comparing presurgery and postsurgery conditions, and classified into categories (Menini et al. 2016). The other study analyzed swelling clinically through a questionnaire and classified it into categories (Yamada et al. 2015).
9. *Presence of calculus* was reported in one study (5.6%), recorded dichotomously (present/absent; Boven et al. 2017).
10. *Gingival index* (peri‐implant inflammation) was reported in 1 study (5.6%), scored 0–3 based on clinical inflammation severity (Boven et al. 2017).
11. *Peri‐implantitis* was reported in 1 study (5.6%), defined as bone loss > 3 mm with bleeding or suppuration (Zhang et al. 2016).
12. *Accepted prosthodontic maintenance events* were analyzed clinically in one study (5.6%), defined as no more than two replacements of patrices, matrices, or matrix components in the first year and no more than five in 5 years (Bouhy et al. 2023).
13. *Prosthesis failure*, defined as more than two repeated fractures of the overdenture necessitating metallic reinforcement, was analyzed clinically in one study (5.6%; Bouhy et al. 2023).
14. *Articulation* was analyzed clinically in one study (5.6%), using a picture naming test of images covering sounds. Digitally recorded samples were rated by two speech‐language therapists (Fonteyne et al. 2019).
15. *Oromyofunctional behavior* was analysed clinically in one study (5.6%), including analysis of the tongue, jaw, lip, and facial muscle movements, as well as blowing and whistling. A questionnaire checked for sucking habits, mouth breathing, lip incompetence, drooling, nail‐biting, and bruxism (Fonteyne et al. 2019).
16. *Accuracy of implant positioning* was reported in one study (5.6%), based on CBCT‐measured deviation (2D, 3D, angular; Pomares‐Puig et al. 2023).
17. *Insertion torque* was reported in one study (5.6%), measured using an implant insertion micromotor (Yamada et al. 2015).
18. *Surgical time* was reported in one study (5.6%), registered during the surgery for implant placement (Yamada et al. 2015).

Additional information on the ClinROs and CROMs is presented in Table 4, with references for the methods cited in the respective studies found in Table S3.

**TABLE 4:** Clinician‐reported outcomes.

Study	Timing of implant placement	Clinician‐reported outcome measures (CROMs)	Methods	Interpretation	Analysis metric	Method of aggregation	Time point	Pre‐/Post‐intervention analysis (ClinROs/CROMs)
Bouhy et al. (2023)	Delayed	Prosthodontic outcomes – Prosthetic complications Prosthodontic success Acceptable prosthodontic maintenance events Prosthesis failure (repeated fractures of the overdenture for which the installation of a metallic reinforcement was then necessary) Implant survival Sulcular Modified Bleeding Index Plaque Index Probing depth	**Prosthodontic outcomes – Prosthetic complications:** Assessment of patrix unscrewing, fracture, loss, or replacement due to significant wear; Matrix fracture or replacement; Dislocation, loss and wear of the matrix component (female nylon inserts); Overdenture maintenance such as fracture, puncture, reline, or prosthesis modification necessary to reposition a new matrix after replacement of a failing implant **Acceptable prosthodontic maintenance events:** Clinical evaluation **Prosthesis failure (repeated fractures of the overdenture for which the installation of a metallic reinforcement was then necessary):** Clinical evaluation **Implant survival:** Clinical evaluation **Sulcular Modified Bleeding Index:** Clinical evaluation. The reference Mombelli et al. (1987), was cited for the methods **Plaque Index:** Clinical evaluation. The reference Löe & Silness (1963) was cited for the methods **Probing depth:** “probing depth in the mesial, distal, lingual and buccal aspects of each implant”	**Prosthodontic outcomes – Prosthetic complications:** NA **Prosthodontic success:** No evidence of retreatment beyond accepted prosthodontic maintenance events **Acceptable prosthodontic maintenance events:** Defined as no more than two replacements of the patrices, matrices or the matrix component during the first year and no more than five replacements in 5 years. The replacement of worn or teeth/fractured overdentures or relining no more than once in 5 years was also considered a success. If the maintenance events exceeded the above‐mentioned criteria, it was considered a prosthodontic complication **Prosthesis failure:** The prosthesis presenting more than two repeated fractures of the overdenture for which the installation of a metallic reinforcement was then necessary was considered a failure **Implant survival:** Implants placed that remained in function and showed no signs of mobility at follow‐up. Implant loss was classified as a failure and directly influenced survival outcomes **Sulcular Modified Bleeding Index:** Score 0: No bleeding when a periodontal probe is passed along the gingival margin adjacent to the implant; Score 1: Isolated bleeding spots visible; Score 2: Blood forms a confluent red line on the margin; Score 3: Heavy or profuse bleeding **Plaque Index: Score** 0 = No plaque in the gingival area; Score 1 = A film of plaque adhering to the free gingival margin and adjacent area of the tooth. The plaque may only be recognized by running a probe across the tooth surface; Score 2 = Moderate accumulation of soft deposits within the gingival pocket, on the gingival margin and/or adjacent tooth surface, which can be seen by the naked eye; Score 3 = Abundance of soft matter within the gingival pocket and/or on the gingival margin and adjacent tooth surface **Probing depth:** NA	Point estimates at each time point	**Prosthodontic outcomes—Prosthetic complications:** Frequency of events **Prosthodontic success:** Frequency of events **Acceptable prosthodontic maintenance events:** Frequency of events **Prosthesis failure (repeated fractures of the overdenture for which the installation of a metallic reinforcement was then necessary):** Frequency of events **Implant survival:** Proportion of surviving implants **Sulcular Modified Bleeding Index:** Frequency distribution (0–3) **Plaque Index:** Frequency distribution (0–3) **Probing depth:** Proportion of sites with probing depth ≥ 6 mm	**Prosthodontic outcomes—Prosthetic complications:** 1, 3, and 5 years after implant placement **Prosthodontic success:** 1, 3, and 5 years after implant placement **Acceptable prosthodontic maintenance events:** 1, 3, and 5 years after implant placement **Prosthesis failure (repeated fractures of the overdenture for which the installation of a metallic reinforcement was then necessary):** 1, 3, and 5 years after implant placement **Implant survival:** 1, 3, and 5 years after implant placement **Sulcular Modified Bleeding Index:** 1, 3, and 5 years after implant placement **Plaque Index:** 1, 3, and 5 years after implant placement **Probing depth:** 5 years after implant placement	**Prosthodontic outcomes – Prosthetic complications:** Post‐intervention analysis **Prosthodontic success:** Post‐intervention analysis **Acceptable prosthodontic maintenance events:** Post‐intervention analysis **Prosthesis failure (repeated fractures of the overdenture for which the installation of a metallic reinforcement was then necessary):** Post‐intervention analysis **Implant survival:** Post‐intervention analysis **Sulcular Modified Bleeding Index:** Post‐intervention analysis **Plaque Index:** Post‐intervention analysis **Probing depth:** Post‐intervention analysis
Boven et al. (2017)	Delayed	Implant survival Peri‐implant bone level changes Modified plaque Index Presence of calculus Peri‐implant inflammation Sulcular Modified Bleeding Index Probing depth	**Implant survival:** Clinical evaluation **Peri‐implant bone level changes:** Assessed by linear measurements on digital panoramic radiographs. Mesial and distal sites were measured, and changes were calculated as the difference between the radiograph taken immediately after implant placement and the one taken 5 years later **Modified Plaque Index:** Clinical evaluation. The reference Mombelli et al. (1987), was cited for the methods **Presence of calculus:** Clinical evaluation **Peri‐implant inflammation (gingival index):** Clinical evaluation. The reference Loe and Silness et al. (1963) (Gingival index) **Sulcular Modified Bleeding Index:** Clinical evaluation. The reference Mombelli et al. (1987), was cited for the methods **Probing depth:** “After removal of the bar, probing depth was measured at four sites of each implant (mesially, labially, distally and lingually) using a periodontal probe (Merrit‐B, Hu‐Friedy). The distance between the marginal border of the mucosa and the tip of the periodontal probe was scored as the probing depth”	**Implant survival**: Implants in function without signs of mobility **Peri‐implant bone level changes**: Negative values indicated bone loss, whereas positive values indicated bone gain **Modified Plaque Index**: Score 0: No detection of plaque; Score 1: Plaque can be detected by running a probe across the smooth marginal surface of the abutment and implant; Score 2: plaque can be seen by the naked eye; Score 3: abundance amount of plaque **Presence of calculus**: Score 1: Presence of calculus; Score 0: Absence of calculus **Peri‐implant inflammation** (gingival index): Score **0:** Absence of inflammation; Score **1:** Mild inflammation—slight change in color and little change in texture; Score **2:** Moderate inflammation—moderate glazing, redness, oedema, and hypertrophy. Bleeding on pressure. Score **3:** Severe inflammation—marked redness and hypertrophy. Tendency to spontaneous bleeding. Ulceration **Sulcular Modified Bleeding Index:** Score 0: No bleeding when a periodontal probe is passed along the gingival margin adjacent to the implant; Score 1: Isolated bleeding spots visible; Score 2: Blood forms a confluent red line on the margin; Score 3: Heavy or profuse bleeding Probing depth: NA	**Implant survival:** Point estimates at each time point **Peri‐implant bone level changes:** Change from the previous time point **Modified plaque Index:** Point estimates at each time point **Presence of calculus:** Point estimates at each time point **Peri‐implant inflammation:** Point estimates at each time point **Bleeding index:** Point estimates at each time point	**Implant survival:** Proportion of surviving implants **Peri‐implant bone level changes:** Median (IQR) of mesial and distal sites, along with frequency distribution across bone loss categories: 0–0.5 mm, > 0.5–1.0 mm, > 1.0–1.5 mm, > 1.5–2.0 mm, > 2.0 mm, and missing data/implant loss **Modified plaque Index:** Median score (IQR) **Presence of calculus:** Median score (IQR) **Peri‐implant inflammation (gingival Index):** Median score (IQR) **Sulcular Modified Bleeding Index:** Median score (IQR) **Probing depth:** Mean (SD)	5 years after implant placement	**Implant survival:** Post‐intervention analysis **Peri‐implant bone level changes:** Post‐intervention analysis **Modified plaque Index:** Post‐intervention analysis **Presence of calculus:** Post‐intervention analysis **Peri‐implant inflammation (gingival Index):** Post‐intervention analysis **Sulcular Modified Bleeding Index:** Post‐intervention analysis **Probing depth:** Post‐intervention analysis
Erkapers et al. (2017)	Delayed	Prosthetic complications	**Prosthetic complications:** Fractured denture tooth; inaccurate seating of angled titanium cylinder; fractured resin provisional bridge; excessive occlusal contacts; food impaction; framework fracture; abutment fracture; abutment loose; phonetic problems; irregularities; bridge screw loosening; construction too bulky	**Prosthetic complications:** NA	Point estimates at each time point	**Prosthetic complications:** Frequency of events	3 and 6 months, 1, 2, and 3 years	**Prosthetic complications:** Post‐intervention analysis
Fonteyne et al. (2019)	Delayed	Articulation Oromyofunctional behavior	**Articulation**: Clinical evaluation. The evaluation used a picture naming test with 135 images of common subjects and actions, covering all Dutch single sounds and most consonant clusters in all permissible syllable positions. Samples were digitally recorded with a video camera. Phonetic inventory and analysis were conducted, with a sound included if produced at least twice. Two speech‐language therapists independently rated the samples **Oromyofunctional behavior:** Clinical evaluation. Patients performed oral muscle tasks to evaluate tongue, jaw, lip, and facial movements. The protocol included tongue positions and movements, jaw and lip movements, facial muscles, and integrated movements like blowing and whistling. Swallowing water and saliva assessed tongue position and lip tension. Behaviors were video recorded and rated on a three‐point scale (0 = “normal”, 1 = “disturbed”, 2 = “impossible”). A questionnaire verified the presence of sucking habits, mouth breathing, lip incompetence, drooling, nail‐biting, and bruxism	**Articulation:** Classification into eight different definitions according to the articulation issues: (1) /s/stridens (1) +/s/simplex (2); (2) Small jaw opening; (3) /z/stridens (1) +/z/simplex (2); (4) /t/interdental (1) + /t/addental (2); (5) /n/interdental; (6) /l/interdental; (7) /ʃ/stridens (1) +/ʃ/simplex (2); (8) /ʒ/stridens (1) +/ʒ/simplex (2) **Oromyofunctional behavior:** Classification into 25 different functions according to the oromyofunctional behavior. For example: Immobility of the jaw, problems with clicking of the tongue, whistling problems, tongue thrust during swallowing, tongue lift problems	Point estimates at each time point	**Articulation:** Mean (SD) number of issues per person. **Oromyofunctional behavior:** Mean (SD) number of issues per person	Beseline (before implant placement), provisional loading, final connection (6 months after implant placement)	**Articulation:** Pre−/post‐intervention analysis **Oromyofunctional behavior:** Pre−/post‐intervention analysis
Menini et al. (2016)	Some immediate and some delayed	Swelling	**Swelling:** Clinical evaluation. Frontal and side images were taken with standardized conditions. Swelling data for both sides of the mouth were recorded. The degree was classified as none, slight, moderate or severe, determined by comparing presurgery and postsurgery photographs	**Swelling:** Classified into four categories: none, slight, moderate, or severe	Point estimates at each time point	**Swelling:** Frequency of events in each category: none, slight, moderate, and severe	Assessed 48 h after the surgery	**Swelling:** Post‐intervention analysis
Pomares‐puig et al. (2023)	Some immediate and some delayed	Accuracy variables (implant position deviation)	**Accuracy variables (implant position deviation):** Accuracy variables were obtained by overlapping preoperative and postoperative CBCT scans using EvaluNav software (ClaroNav). The software automatically detected implant positions and calculated deviations in platform (3D and 2D), apex (3D and depth), and angle. The postoperative CBCT was acquired after implant placement, before abutment connection	**Accuracy variables (implant position deviation):** Deviation between planned and actual implant position (platform, apex, depth, angle)	Point estimates of deviation	**Accuracy variables (implant position deviation):** Mean (SD)	After implant placement (before abutment connection)	**Accuracy variables (implant position deviation):** Post‐intervention analysis
Testori et al. (2021)	Immediate	Implant survival Prosthetic survival	**Implant survival:** Clinical evaluation **Prosthetic survival:** Clinical evaluation	**Implant survival:** Implant present and functional **Prosthetic survival:** Prosthesis in function	Point estimates at each time point	**Implant survival:** Proportion of surviving implants **Prosthetic survival:** Proportion of surviving prostheses	1 year and 8 months (20 months)	**Implant survival:** Post‐intervention analysis **Prosthetic survival:** Post‐intervention analysis
Van Doorne et al. (2020)	Delayed	Implant survival Prosthetic survival	**Implant survival:** Clinical evaluation **Prosthetic survival:** Clinical evaluation	**Implant survival:** Implant present and functional **Prosthetic survival:** Prosthesis in function	Point estimates at each time point	**Implant survival:** Proportion of surviving implants **Prosthetic survival:** Proportion of surviving prostheses	6 months, 1 and 2 years	**Implant survival:** Post‐intervention analysis **Prosthetic survival:** Post‐intervention analysis
Yamada et al. (2015)	Delayed	Insertion torque Surgical time Implant survival. Prosthetic survival. Peri‐implant bone level changes Post operative swelling Complications (surgical, with the provisional and postoperative)	**Insertion torque:** Measured using an implant insertion micromotor (GC Implant Motor IM‐III, GC Corp), which was able to measure torque between 0 and 70 Ncm in increments of 5 Ncm **Surgical time:** Registration of the time between placement of the surgical template in the patient's maxilla and removal of the surgical template after implant placement **Implant survival:** Clinical evaluation **Prosthetic survival:** Clinical evaluation **Peri‐implant bone level changes:** Standardized intraoral radiographs were taken at implant placement, 6 months, and 12 months using a customized indicator made for each implant with a silicone index. The known distance between the implant's microthreads (0.4 mm) was used as a reference to determine the marginal bone level on the mesial and distal surfaces. Measurements were rounded to the nearest 0.1 mm to calculate bone level changes over time. **Swelling:** Clinical evaluation. A questionnaire with a scale where: 0 = no visible swelling; 1 = mild to moderate swelling visible in the mouth; 2 = moderate visible facial swelling; 3 = excessive swelling up to the orbital border; and 4 = severe swelling affecting the eyelid, facial muscles, and swallowing **Complications:** Surgical complications, including misfit of the surgical guide; misfit of the occlusal index; limited oral aperture; fracture of the surgical guide; absence of primary stability; bone perforation; Provisional complications, including misfit of the provisional prosthesis; major occlusal adjustments. Postoperative complications, including abutment screw loosening; fracture of the provisional prosthesis; mobility of implant	**Insertion torque**: NA **Surgical time:** NA **Implant survival:** An implant was considered surviving if it was still present without signs of mobility or discomfort, symptoms of neurologic problems, or signs of peri‐implantitis **Prosthetic survival:** The superstructure was defined as surviving if there had been no remake of the definitive prosthesis **Peri‐implant bone level changes:** Negative values indicated bone loss, whereas positive values indicated bone gain **Swelling:** As the score increases from 0 to 4, the severity and extent of swelling become more pronounced and impact larger areas and functions **Complications:** NA	**Insertion torque:** Point estimates at each time point **Surgical time:** Point estimates at each time point **Implant survival:** Point estimates at each time point **Prosthetic survival:** Point estimates at each time point **Peri‐implant bone level changes:** Change from the previous time point **Swelling:** Point estimates at each time point **Complications:** Point estimates at each time point	**Insertion torque:** Mean (SD) **Surgical time:** Mean (SD) **Implant survival:** Proportion of surviving implants **Prosthetic survival:** Proportion of surviving prostheses **Peri‐implant bone level changes:** Mean (SD) of mesial and distal sites **Swelling:** Frequencies (0–4). **Complications:** Frequency of events	**Insertion torque:** During the surgery for implant placement **Surgical time:** During the surgery for implant placement **Implant survival:** 1 year **Prosthetic survival:** 1 year **Peri‐implant bone level changes:** At implant placement, 6 months and 1 year **Swelling:** Immediately after implant placement and immediate loading **Complications:** At implant placement, 6 months and 1 year	**Insertion torque:** Intraoperative **Surgical time:** Intraoperative **Implant survival:** Post‐intervention analysis **Prosthetic survival:** Post‐intervention analysis **Peri‐implant bone level changes:** Post‐intervention analysis **Swelling:** Post‐intervention analysis **Complications:** Post‐intervention analysis
Zhang et al. (2016)	Delayed	Implant success Prosthetic success Prosthetic–Technical complications Modified Plaque Index Probing depth Sulcular Modified Bleeding Index Peri‐implant bone level changes—Marginal bone loss Peri‐implantitis	**Implant success:** Clinical evaluation **Prosthetic success:** Clinical evaluation **Prosthetic—Technical complications:** Veneer ceramic chipping, loss of retention, abutment loosening, fracture of framework, abutment, screw, and implant **Modified Plaque Index:** Clinical evaluation. The reference Mombelli et al. (1987) was cited for the methods of this outcome. For all peri‐implant clinical assessments, a periodontal probe (15 UNC/CP‐11.5B Screening Color‐Coded Probe, Hu‐Friedy) was used **Probing depth:** Clinical evaluation. The reference Mombelli et al. (1987) was cited for the methods of this outcome. For all peri‐implant clinical assessments, a periodontal probe (15 UNC/CP‐11.5B Screening Color‐Coded Probe, Hu‐Friedy) was used **Sulcular modified bleeding index:** Clinical evaluation. The reference Mombelli et al. (1987) was cited for the methods of this outcome. For all peri‐implant clinical assessments, a periodontal probe (15 UNC/CP‐11.5B Screening Color‐Coded Probe, Hu‐Friedy) was used **Peri‐implant bone level changes—Marginal bone loss:** Standardized intraoral radiographs were obtained using a Rinn film holder with beam alignment perpendicular to the implant. Images were scanned and analyzed using SIDEXIS software by a blinded examiner. Marginal bone level was measured from the implant collar to the most coronal bone‐to‐implant contact at mesial and distal sites, and bone loss was calculated as the mean of both sites **Peri‐implantitis:** Peri‐implantitis was defined as marginal bone loss exceeding 3 mm in combination with bleeding on probing or suppuration, or both	**Implant success:** The implant success criteria were determined by: no clinically detectable implant mobility; no pain or any subjective sensation; no recurrent peri‐implant infection and no continuous radiolucency around the implant **Prosthetic success:** A prosthesis was regarded as a success if it remained unchanged and no intervention was necessary during the time of observation, while those which had to be replaced with new prostheses were regarded as failure **Prosthetic–Technical complications:** NA **Modified Plaque Index:** Score 0: No detection of plaque; Score 1: Plaque can be detected by running a probe across the smooth marginal surface of the abutment and implant; Score 2: plaque can be seen by the naked eye; Score 3: abundance amount of plaque **Probing depth:** NA **Sulcular modified bleeding index:** Score 0: No bleeding when a periodontal probe is passed along the gingival margin adjacent to the implant; Score 1: Isolated bleeding spots visible; Score 2: Blood forms a confluent red line on the margin; Score 3: Heavy or profuse bleeding **Peri‐implant bone level changes—Marginal bone loss:** Bone loss relative to the previous time point was reported as a positive value **Peri‐implantitis:** NA	**Implant success:** Point estimates at each time point **Prosthetic success:** Point estimates at each time point **Technical complications:** Point estimates at each time point **Modified Plaque Index:** Point estimates at each time point **Probing depth:** Point estimates at each time point **Sulcular Modified Bleeding Index:** Point estimates at each time point **Peri‐implant bone level changes ‐ Marginal bone loss:** Change from the previous time point **Peri‐implantitis:** Point estimates at each time point	**Implant success:** Proportion of successful implants **Prosthetic success:** Proportion of successful prostheses **Prosthetic– Technical complications:** Frequency of events by category **Modified Plaque Index:** Frequency distribution (scores 0–3) **Probing depth:** Mean (SD) **Sulcular Modified Bleeding Index:** Frequency distribution (scores 0–3) **Peri‐implant bone level changes ‐ Marginal bone loss:** Mean (SD) of mesial and distal sites **Peri‐implantitis:** Frequency of diagnosed cases	**Implant success:** 1, 3, 5, and 10 years after prosthesis delivery **Prosthetic success:** 1, 3, 5, and 10 years after prosthesis delivery **Prosthetic ‐ Technical complications:** 1, 3, 5, and 10 years after prosthesis delivery **Modified Plaque Index:** 1, 3, 5, and 10 years after prosthesis delivery **Probing depth:** At the prosthesis delivery and 1, 3, 5, and 10 years after prosthesis delivery **Sulcular Modified Bleeding Index:** At the prosthesis delivery and 1, 3, 5, and 10 years after prosthesis delivery **Peri‐implant bone level changes—Marginal bone loss:** At the prosthesis delivery and 1, 3, 5, and 10 years after prosthesis delivery **Peri‐implantitis:** 1, 3, 5, and 10 years after prosthesis delivery	**Implant success:** Post‐intervention analysis **Prosthetic success:** Post‐intervention analysis **Prosthetic—Technical complications:** Post‐intervention analysis **Modified Plaque Index:** Post‐intervention analysis **Probing depth:** Post‐intervention analysis **Sulcular Modified Bleeding Index:** Post‐intervention analysis **Peri‐implant bone level changes – Marginal bone loss:** Post‐intervention analysis **Peri‐implantitis:** Post‐intervention analysis

## 4 Discussion

### 4.1 Main Findings

The aim of the present systematic review was to assess the outcomes and measures used to analyze PROs and ClinROs in prospective clinical studies focusing on the timing of implant placement in fully edentulous maxilla or maxilla with residual dentition scheduled for extraction and requiring implant‐supported prosthesis. The review shows a wide array of PROs (16) and ClinROs (18) used across the 13 included studies, with varying assessment tools and methods. While this diversity demonstrates the tailored nature of outcome measures, it also underscores the importance of harmonizing core outcomes to improve comparability and consistency across studies, which is an essential step in developing a core outcome set (Williamson et al. 2017; Kirkham et al. 2017). Regardless of the timing of implant placement, no clear trend emerged in terms of the outcomes assessed and the analysis methods employed, indicating variability across studies.

The organization of the results in this study was adapted following data extraction due to substantial heterogeneity in the outcomes and measurement methods encountered, which were categorized into PROs/PROMs and ClinROs/CROMs.

### 4.2 PROs and PROMs

The PROs in the included studies covered various aspects of patient experience, such as treatment impact on quality of life and satisfaction with treatment and oral health. They also assessed surgery‐related outcomes, including pain intensity, postoperative drug use, everyday life impairment, patient well‐being, and comfort. Additionally, outcomes related to implant‐supported prostheses, such as satisfaction, functional complaints, and masticatory function, were evaluated. Within each of these main areas, the outcomes and assessment methods varied significantly.

Some studies focused on specific aspects such as speech, eating ability, and psychosocial impact, while others provided a more general assessment of quality of life and satisfaction. This variety reflects the comprehensive nature of PROs but also points to the need for a consensus on essential PROs (Williamson et al. 2017). Establishing a minimum core outcome set would ensure that key PROMs are consistently included in trials within this area, facilitating more standardized and comparable results (Kirkham et al. 2017).

Oral health‐related quality of life was the most assessed outcome of PROMs. Different versions of the Oral Health Impact Profile (OHIP) were used for this purpose, including OHIP‐14, OHIP‐20, OHIP‐49, and OHIP‐J49. OHIP‐49, with its 49 items, is the original and most comprehensive version, covering a wide range of issues related to oral health and its impact on quality of life (Slade and Spencer 1994). It offers in‐depth insight into the seven domains (functional limitation, physical pain, psychological discomfort, physical disability, psychological disability, social disability, and handicap; Slade and Spencer 1994), but can be time‐consuming to administer. The OHIP‐14 was the most used instrument in the included studies. Comprising 14 items, it is a highly abbreviated version of the OHIP‐49, focusing on the most critical aspects of oral health‐related quality of life (Slade 1997). It retains the seven original domains but only two items per domain, making it quick to administer but potentially less comprehensive. However, OHIP‐14 may have compromised measurement properties for edentulous patients due to the exclusion of items related to chewing and denture‐wearing during its development (Locker et al. 2001; Allen and Locker 2002). Additionally, it may experience floor effects, where many participants score at the lower end of the scale (Bindman et al. 1990). This occurs because the instrument lacks sensitivity at the lower end, often due to the exclusion of relevant items (Locker et al. 2001; Allen and Locker 2002). As a result, it can underestimate the true extent of issues or changes in edentulous patients' oral health status. In contrast, OHIP‐20, dedicated to edentulous patients, includes questions on chewing difficulty, food catching, denture fitting, and others, addressing specific needs (Allen and Locker 2002). Standardizing certain OHIP versions is important, as using different versions across studies complicates result comparison.

VAS, NRS, and VRS were frequently used to measure pain intensity. The VAS is statistically robust and provides ratio‐level data, but it is the most challenging to use and has the highest failure rate (Williamson and Hoggart 2005; Karcioglu et al. 2018). Elderly patients and individuals with cognitive impairments and communication difficulties often found verbal descriptors or rating scales more practical for expressing pain intensity (Karcioglu et al. 2018). Both the VAS and NRS are highly sensitive in detecting changes in pain levels. The NRS offers interval‐level data, is easy to administer, and is more suitable for pain assessment, audits, and research. Although the VRS is the least sensitive, it is the simplest to use (Williamson and Hoggart 2005; Karcioglu et al. 2018). However, all three scales are valid, reliable, and appropriate for clinical practice (Williamson and Hoggart 2005; Karcioglu et al. 2018). Despite their validity and reliability, the specific implementations of these scales varied, leading to inconsistent reporting. Additionally, there was significant variation in the interpretation of the results, with different studies using varying levels, categories, and thresholds. Methods of reporting also differed, with some studies presenting the variables as categories and frequencies while others used continuous variables.

Patient satisfaction was also evaluated in the included studies, and VAS was the most used tool. Measuring patient satisfaction is challenging due to its complex definition and the influence of confounders, which can be patient‐ or measurement‐related (Voutilainen et al. 2016). Despite its limitations, the VAS is sensitive and appropriate for this measurement. It demonstrates reduced susceptibility to the ceiling effect, which occurs when respondents frequently achieve the highest possible score, limiting the scale's ability to capture the full range of variability (Voutilainen et al. 2016).

The most common baseline for evaluating PROs was before implant placement. However, subsequent time points varied depending on the outcomes, even for the same outcomes. For oral health‐related quality of life using different OHIP questionnaires, the second evaluation ranged from 1 week to 1 year after surgery. Pain was analyzed intraoperatively (immediately after surgery) and postoperatively (within the first 2 days or 1 week after surgery). Patient satisfaction was primarily assessed before implant placement, with follow‐up periods ranging from the time of provisional prosthesis delivery (1 week after surgery) to 5 years after implant placement. This variability in follow‐up times can challenge the analysis and interpretation of PROs, potentially affecting the consistency and comparability of the results.

Clear differences were observed not only in the selected outcomes but also in the assessment tools and timing used for similar PROs. This variability hinders result comparisons across studies and underscores the need for standardized PROs in implant dentistry. Diverse tools and methods for assessing and interpreting similar outcomes challenge meaningful comparisons and data synthesis. This heterogeneity leads to inconsistencies in reported outcomes, making it difficult to identify the most effective clinical practices from the patient's perspective.

### 4.3 ClinROs and CROMs

Ten of the included studies assessed ClinROs, encompassing a broad range of clinical outcomes, including traditional clinical parameters on peri‐implant health and implant stability, surgical parameters, function, and prosthetic performance. ClinROs also exhibited considerable variability in the methods and domains assessed.

The most frequently reported were implant survival and prosthetic complications. Implant survival was evaluated in five studies, generally defined as the implant being in place without mobility, with one study also requiring the absence of discomfort, pain, or neurologic symptoms. Although this outcome is fundamental in implant dentistry, heterogeneity in its definition and the lack of standardized follow‐up protocols may hinder direct comparisons (Papaspyridakos et al. 2012). Prosthetic complications and prosthetic survival were the second most commonly reported ClinROs, covering a wide spectrum of mechanical and technical issues. Inconsistent categorization and varying definitions for what constitutes a “complication” across studies pose challenges for synthesis (Papaspyridakos et al. 2012).

Other traditional ClinROs, such as peri‐implant bone level changes, probing depth, plaque accumulation, presence of calculus, and bleeding indices, were also reported. These are indicators of peri‐implant health and implant stability. However, differences in radiographic methods (e.g., standardized intraoral vs. panoramic imaging; Dos Reis et al. 2025), clinical techniques (e.g., probe type and measurement method; Monje and Salvi 2024), and data aggregation and reporting approaches may affect measurement accuracy and comparability.

Peri‐implantitis incidence was assessed in one study. As a composite outcome, it requires standardized diagnostic parameters to enable consistent and comparable incidence estimates across studies. In this sense, the case definitions for peri‐implant diseases should follow those established in the 2017 World Workshop (Berglundh et al. 2018; Tonetti et al. 2023).

The accuracy of implant positioning, assessed via CBCT superimposition, was reported in one study involving immediate implant placement and loading (Pomares‐Puig et al. 2023), highlighting a gap in the literature regarding the precision of surgical execution in such cases.

Insertion torque and surgical time, two intraoperative outcomes, were reported in one study (Yamada et al. 2015). Insertion torque reflects primary implant stability and is influenced mainly by implant design and bone quality (Hadaya et al. 2022). Surgical time provides an indirect measure of procedural complexity and operator efficiency. While both are valuable for intraoperative assessment, they remain underreported.

Finally, the inclusion of outcomes such as articulation, oromyofunctional behavior, and swelling reflects an effort to capture broader functional aspects of rehabilitation. However, these outcomes were rarely reported, and typically by single studies. Future studies should also focus on these outcomes, as the clinician's subjective perspective is crucial in defining the success of implant procedures and guiding treatment plans and interventions. Moreover, ClinROs can complement PROs by offering a clinical perspective that balances the patient's subjective experiences (Powers et al. 2017).

Postsurgical swelling was assessed in two of the four studies using standardized frontal and side images and a clinical examination using questionnaires, both classifying swelling into categories. While swelling is an important indicator of postoperative inflammation (Antonelli et al. 2023), the heterogeneity in assessment methods (images vs. questionnaires) impairs comparability across studies.

The ClinROs related to the prosthesis were derived from one study, which defined criteria for acceptable prosthodontic maintenance events, prosthetic complications, and prosthesis failure. These criteria provide a framework for evaluating the long‐term success and durability of implant‐supported prostheses. By establishing clear thresholds for acceptable maintenance and complications, clinicians can better manage patient expectations and improve treatment planning. This focus is also crucial because frequent interventions can lead to increased patient discomfort, higher costs, and reduced overall satisfaction (Karimbux et al. 2023). However, more comprehensive research and validation of these criteria are needed, as they currently rely on a single study. A clear and standardized definition for complications and failures for both prostheses and implants would be beneficial for future studies (Papaspyridakos et al. 2012).

Regarding articulation and oromyofunctional behavior, both outcomes were assessed in one study. Articulation, speech clarity, and quality are relevant aspects of patient quality of life (Allen et al. 2001). Oromyofunctional behavior, a comprehensive evaluation that identifies issues like tongue thrust during swallowing and problems with jaw mobility, is important for ensuring proper oral function and comfort with implant‐supported prostheses (Fonteyne et al. 2019). Despite this, these outcomes were evaluated in only one of the included studies. Other studies have used these methods in different clinical contexts, not specifically for the rehabilitation of the fully edentulous maxilla with implant‐supported prostheses (Van Lierde et al. 2011, 2012).

Clear differences were observed in the selection and assessment of ClinROs, with notable variability in the methods, domains, and criteria applied across studies. Standardized clinical tests and objective measures were often lacking, with many studies relying on subjective clinician evaluations. This reliance can introduce bias and variability, limiting the comparability and reliability of findings (Powers et al. 2017).

### 4.4 Risk of Bias

The included randomized controlled trial showed risk of bias concerns due to two main issues: insufficient information on allocation sequence concealment and the lack of a registered protocol. Allocation sequence concealment prevents selection bias, and a registered protocol ensures transparency and accurate reporting. Without these, it is challenging to verify if the study was conducted and reported as intended (Spieth et al. 2016).

Regarding the included case series, the lowest scores were in three critical areas: complete inclusion of participants (Q5), clear reporting of clinical information (Q7), and outcomes or follow‐up results (Q8). These low scores indicate significant gaps in the transparency and completeness of the data presented (Martin 2017). The low score in Q5 suggests that not all eligible participants were included, potentially introducing selection bias. Poor reporting of clinical information (Q7) can obscure important details about the patient population and their treatment, which could influence the outcomes, including PROMs and CROMs. Inadequate reporting of outcomes or follow‐up results (Q8) hinders the ability to evaluate the effectiveness and long‐term outcomes (Mayo‐Wilson et al. 2017; Souza et al. 2023).

Comprehensive outcome reporting, ideally following the CONSORT guidelines (Schulz et al. 2010) and the five levels of outcome reporting as outlined in the SPIRIT statement (Chan et al. 2013; Calvert et al. 2018), is essential for accurately evaluating the success and durability of interventions. An outcome is fully defined when it encompasses five levels: (1) domain, (2) specific measure, (3) specific metric, (4) aggregation method, and (5) time point. Incomplete specification of outcomes in the protocol or publication can lead to selective reporting, where authors may choose measures, metrics, and time points that yield statistically significant results (Mayo‐Wilson et al. 2017; Souza et al. 2023).

### 4.5 Strengths and Limitations

Some limitations of this review should be considered. When applying all the eligibility criteria, no comparative studies regarding the timing of implant placement in the maxilla were found. As a result, this topic was not the primary focus of the included studies, most of which were single‐arm studies. Additionally, the review was limited to manuscripts published in English. Restricting the review to manuscripts published in English aimed to maintain consistency in reporting and interpretation, though it resulted in the exclusion of one study published in another language (Table S2).

This review adopted the consensus definition of immediate implant placement, specifying it as occurring at the time of extraction or within 10 days (Tonetti et al. 2019). However, it is acknowledged that clinical outcomes and patient experiences associated may differ significantly between same‐day placement and placement occurring up to 10 days post‐extraction. An alternative, perhaps more contextually appropriate definition describes immediate implant placement as performed immediately after tooth extraction within the same surgical procedure (Chen et al. 2004; Hämmerle et al. 2004; Gallucci et al. 2018). Notably, all studies included in this review that adhere to an immediate protocol involved implant placement during the same surgical procedure as the extraction. These timing variations may impact PROs/PROMs and ClinROs/CROMs, such as pain and swelling, and should therefore be carefully considered when selecting outcomes to be assessed.

Different loading protocols (immediate, early, or delayed) may also influence PROs/PROMs and ClinROs/CROMs, and information on loading timing has been provided for each study to offer context (Gallucci et al. 2018). However, this review specifically addresses outcomes related to implant placement timing, while a separate systematic review assessed outcomes associated with implant loading timing.

Additionally, although bone augmentation was performed in a subset of patients in four of the included studies, its specific impact on PROs/PROMs and ClinROs/CROMs was not evaluated.

The strengths of this review include the diverse range of outcomes identified and the comprehensive search strategy implemented, covering five major databases along with detailed hand searching. The data from this systematic review can be used for the establishment of a core outcome set in the field of rehabilitation of maxillary edentulous patients. Rather than evaluating the effectiveness of individual outcomes, this review aims to identify existing tools and facilitate an evidence‐based consensus process to guide the selection of standardized outcomes for future studies.

### 4.6 Future Perspectives

For future research, integrating both PROs and ClinROs should be considered for a comprehensive understanding of treatment efficacy and patient satisfaction (Kirkham et al. 2017). Developing a core set of PROMs and CROMs should be a priority to improve the selection and reporting of these outcomes, enhancing the comparability and utility of findings, and making them more actionable for clinical practice (Kirkham et al. 2017).

Moreover, it is important to consistently report contextual factors of the treatment that may influence PROs, such as whether treatments are provided under subsidized or research‐based settings, which may or may not cover patient costs. Treatment expenses and the perceived cost‐effectiveness can influence patient perceptions and subsequently affect reported outcomes (Dean et al. 2021; Bianchim et al. 2023). Additional biases in PROs can arise when perceived satisfaction is influenced more by the treatment setting than by clinical outcomes alone. Influential factors include patient‐related influences (like trust in the professional team, social desirability, and expectations shaped by pre‐treatment counseling or awareness of other patient outcomes) and treatment‐specific effects (such as the innovative nature of the treatment and placebo responses). To mitigate these biases, future studies should employ strategies like blinding patients to intervention type, center, and professional reputation; using control groups, and assessing baseline expectations. Consistently reporting these fundamental characteristics will enhance the interpretability and comparability of PROs across diverse treatment settings (Roydhouse et al. 2019; Kluzek et al. 2022).

It is essential to emphasize that PROMs should be validated for the specific conditions and outcomes they are intended to measure. These instruments can be either general or condition‐specific. General PROMs assess broad aspects related to a variety of health conditions, but they may lack sensitivity to detect the specific symptoms of a condition, such as complete edentulism, which could influence treatment options and outcomes. In contrast, condition‐specific instruments are designed to focus on particular symptoms and their impact on the patient's health, making them more capable of detecting and quantifying specific symptoms both cross‐sectionally and over time related to that condition (Leles et al. 2022; Needleman et al. 2023).

In the context of implant treatment for complete edentulism in the maxilla, the instruments should be adapted and validated for this unique clinical scenario. This differs from assessing partial tooth loss or areas generally considered to have less aesthetic relevance in this context, such as the mandible. Therefore, PROMs must account for the clinical characteristics of edentulous patients and the effects of different treatment options, such as dental implants. The use of validated instruments specifically designed for this context would allow for a more precise assessment of patient outcomes. There should be a preference for using validated instruments that are tailored to the clinical context of interest, and further validation for implant placement specifically would enhance their applicability.

The validation of PROMs is a key process in ensuring their appropriateness for measuring the specific outcomes in a given context (McKenna 2011). As shown in Table S3, some studies conducted a formal validation process for the specific PROM, testing reliability, validity, and sensitivity, thereby confirming the tool's suitability for assessing the desired condition (i.e., OHIP‐14 for oral health‐related quality of life). However, in other cases, although the PROM was used in referenced studies, no formal validation was conducted. Instead, some studies relied on previous research where the PROM was adopted without specific validation. Additionally, there were instances where the PROM was used without any previous formal validation or reference to prior studies, suggesting the tool was applied based on the authors' judgment or adapted for the specific study. Beyond general PROM validation, a limited number of studies conducted validation specifically within the context of implant treatment. Therefore, the use of validated tools should be prioritized, ideally those specifically validated for the clinical context in which they will be applied, such as in implant treatments, as previously discussed.

The ID‐COSM project (Tonetti et al. 2023), grounded in previously performed systematic reviews, established a core outcome set for implant dentistry, focusing on essential outcome domains. However, it did not address specific PROs and ClinROs, measurement methods, or ideal assessment points for diverse clinical scenarios. The findings of the present review complement the ID‐COSM framework by providing groundwork for a future consensus aimed at defining these specific outcomes and measures, thereby enhancing consistency and precision in studies that will be planned and conducted in the future.

## 5 Conclusions

The PROs and ClinROs reported in the included studies exhibit substantial variability in domains, assessment methods, and reporting practices. These findings highlight the need for harmonization in outcome selection and the development of a core outcome set for PROs/PROMs and ClinROs/CROMs to enhance comparability and reliability across studies.

## Author Contributions

**Giuseppe A. Romito:** conceptualization, writing – review and editing, project administration, writing – original draft, formal analysis. **Isabella Neme Ribeiro dos Reis:** investigation, writing – original draft, methodology, software, data curation, formal analysis, writing – review and editing. **Mohamed A. Hassan:** investigation, writing – original draft, writing – review and editing, methodology, software, formal analysis, data curation. **Cristina Cunha Villar:** writing – review and editing, supervision. **Helena Francisco:** writing – review and editing. **Claudio Mendes Pannutti:** conceptualization, writing – review and editing, methodology.

## Conflicts of Interest

The authors declare no conflicts of interest.

## Supporting information


**Table S1.** Search strategies for each database.

**Table S2.** List of the excluded studies (*N* = 43).

**Table S3.** References for the methods used in the studies.

## Data Availability

The data that support the findings of this study are available from the corresponding author upon reasonable request.
