# Supplementary material for: Patient‐ and Clinician‐Reported Outcomes and Outcome Measures Evaluating Timing of Implant Placement in the Edentulous Maxilla: A Systematic Review of Clinical Studies
Source: Clin Oral Implants Res. 2026 Feb 24;37(Suppl 30):S302–31. doi: 10.1111/clr.14454 (PMC12930140; doi:10.1111/clr.14454)
Supplement: Supplementary file 1 — Table S1. Search strategies for each database. [file CLR-37-S302-s001.docx]

Table S1 – Search strategies for each database

| Databases | |
| --- | --- |
| Pubmed | #1 (jaw, edentulous OR mouth, edentulous OR edentulous OR tooth loss)  AND (maxilla OR upper jaw OR maxillary)  #2 ( Dental Implants, Mini OR dental implant OR implant placement/timing OR  implant performance OR alveolar ridge preservation OR bone grafting  OR implant survival)  #3 (visual analog scale OR quality of life OR patient satisfaction OR  patient reported outcome measure* OR PROM* OR OHIP OR questionnaire* OR  patient centered outcome OR patient-reported outcome OR patient related OR  Clinician satisfaction OR clinician-reported outcomes OR CROM*)  #4 #1 AND #2 AND #3 |
| Embase | #1 ('jaw, edentulous' OR 'mouth, edentulous' OR edentulous OR 'tooth loss' ) AND  (maxilla OR 'upper jaw' OR maxillary )  #2 ( 'Dental Implants, Mini' OR 'dental implant' OR 'implant placement/timing' OR  'implant performance' OR 'alveolar ridge preservation' OR 'bone grafting' OR 'implant survival')  #3 ('visual analog scale' OR 'quality of life' OR 'patient satisfaction' OR  'patient reported outcome measure*' OR ‘PROM*’ OR ‘OHIP’ OR ‘questionnaire*’  OR 'patient centered outcome' OR 'patient-reported outcome' OR 'patient related' OR  'Clinician satisfaction' OR 'clinician-reported outcomes' OR 'crom*')  #4 #1 AND #2 AND #3 |
| Scopus | #1 TITLE-ABS-KEY ( ( "jaw, edentulous" OR "mouth, edentulous" OR edentulous  OR "tooth loss" ) AND ( maxilla OR "upper jaw" OR maxillary ) )  #2 TITLE-ABS-KEY( ( "Dental Implants, Mini" OR "dental implant" OR  "implant placement/timing" OR "implant performance" OR  "alveolar ridge preservation" OR "bone grafting" OR "implant survival" ) )  #3 TITLE-ABS-KEY ( ( "visual analog scale" OR "quality of life" OR  "patient satisfaction" OR  "patient reported outcome measure*" OR prom*  OR ohip OR questionnaire* OR "patient centered outcome" OR  "patient-reported outcome" OR "patient related" OR "Clinician satisfaction" OR  "clinician-reported outcomes" OR crom*) )  #4 #1 AND #2 AND #3 |
| Web of Science | #1 TS=(("jaw, edentulous" OR "mouth, edentulous" OR "edentulous" OR "tooth loss" )  AND ("maxilla" OR "upper jaw" OR "maxillary" ))  #2 TS=("Dental Implants, Mini" OR "dental implant" OR "implant placement/timing"  OR "implant performance" OR "alveolar ridge preservation" OR "bone grafting"  OR "implant survival")  #3 TS=("visual analog scale" OR "quality of life" OR "patient satisfaction" OR  "patient reported outcome measure*" OR PROM* OR OHIP OR questionnaire* OR  "patient centered outcome" OR "patient-reported outcome" OR "patient related"  OR "Clinician satisfaction" OR "clinician-reported outcomes" OR crom*)  #4 #1 AND #2 AND #3 |
| Register | |
| Cochrane Central  Register of Controlled  Trials (CENTRAL) | #1 MeSH descriptor: [Jaw, Edentulous] explode all trees  #2 MeSH descriptor: [Mouth, Edentulous] explode all trees  #3 MeSH descriptor: [Tooth Loss] explode all trees  #4 (jaw edentulous OR mouth edentulous OR edentulous OR tooth loss )  #5 #1 OR #2 OR #3 OR #4  #6 MeSH descriptor: [Maxilla] explode all trees  #7 (maxilla OR upper jaw OR maxillary )  #8 #6 OR #7  #9 #5 AND #8  #10 MeSH descriptor: [Dental Implants] explode all trees  #11 (dental implant OR Dental Prosthesis Implant-Supported OR full-arch )  #12 #10 OR #11  #13 MeSH descriptor: [Visual Analog Scale] explode all trees  #14 ("visual analog scale" OR "quality of life" OR "patient satisfaction" OR  ("patient reported outcome" NEXT measure*) OR PROM* OR OHIP OR questionnaire*  OR "patient centered outcome" OR "patient-reported outcome" OR "patient related" )  #15 #13 OR #14  #16 #9 AND #12 AND #15 |
