# Supplementary material for: Patient‐ and Clinician‐Reported Outcomes and Outcome Measures Evaluating Timing of Implant Placement in the Edentulous Maxilla: A Systematic Review of Clinical Studies
Source: Clin Oral Implants Res. 2026 Feb 24;37(Suppl 30):S302–31. doi: 10.1111/clr.14454 (PMC12930140; doi:10.1111/clr.14454)
Supplement: Supplementary file 2 — Table S2. List of the excluded studies (N = 43). [file CLR-37-S302-s002.docx]

Table S2 – List of the excluded studies (N = 43).

| Study | Reason for exclusion |
| --- | --- |
| Aboelez et al., 2022 | The objective of this study was to compare two types of abutments. |
| Aboelez et al., 2023 | The objective of this study was to compare implants placed in different positions (angles). |
| Abouelhuda et al., 2023 | Zygomatic implants |
| Agliardi et al., 2014 | Article first published in 2012 |
| Agliardi et al., 2017 | The article aimed to compare zygomatic and standard implants. |
| Amorfini et al., 2016 | The study aimed to compare guided and conventional surgery for implant placement |
| Anuradha et al., 2020 | Methods used to analyze patient satisfaction were not presented |
| Balaguer et al., 2015 | PROMs or CROMs were not evaluated |
| Bernard et al., 2019 | The objective of this study was to compare different timings of loading. |
| Bettach et al., 2015 | Article first published in 2013 |
| Browaeys et al., 2014 | PROMs or CROMs were not evaluated |
| Busenlechner et al., 2016 | PROMs or CROMs were not evaluated |
| Caramês et al., 2022 | PROMs or CROMs were not evaluated |
| Chen et al., 2017 | Implant placement in partially edentulous patients |
| Davó et al., 2018 | The article aimed to compare zygomatic and standard implants. |
| Ebinger et al., 2015 | Patients already had the implants placed before the study started. |
| Fayek et al., 2022 | The study aimed to compare conventional dentures and overdentures. |
| Felice et al., 2020 | The study aimed to compare short implants with long implants combined with bone grafts. |
| Fernandes et al., 2023 | Implant placement in partially edentulous patients |
| Fernández-Ruiz et al., 2021 | The study aimed to compare zygomatic implants with standard implants (all-on-four) |
| Ferrer et al., 2020 | The study aimed to compare two types of attachments in overdentures |
| Gong et al., 2023 | The study aimed to compare different contact areas (with the mucosa) |
| Kuoppala et al., 2015 | Patients already had the implants placed before the study started. |
| Li et al., 2017 | Methods used to analyze patient satisfaction were not presented |
| Lie et al., 2024 | The study aimed to compare three groups: natural teeth, sinus lift without graft, and sinus lift with graft. |
| Markovic et al., 2022 | The objective of this study was to compare different timings of loading. |
| Meloni et al., 2016 | PROMs or CROMs were not evaluated |
| Mendes et al., 2016 | Patients already had the implants placed before the study started. |
| Niedermaier et al., 2016 | PROMs or CROMs were not evaluated |
| Nomiyama et al., 2023 | The study aimed to compare guided and conventional surgery for implant placement |
| Onclin et al., 2023 | The study aimed to compare overdentures supported by 2 implants versus 4 implants. |
| Osman et al., 2014 | Article first published in 2012 |
| Papaspyridakos et al., 2014 | Case report |
| Peñarrocha-Oltra et al., 2014 | The objective of this study was to compare different timings of loading. |
| Rignon-bret et al., 2022 | Conventional dentures |
| Rossi et al., 2021 | The study aimed to compare short implants with 10 mm implants plus sinus lift in the posterior region |
| Slot et al., 2014 | Article first published in 2012 |
| Slot et al., 2022 | The study aimed to compare prostheses supported by different numbers of implants. |
| Slot et al., 2022 | The study aimed to compare prostheses supported by different numbers of implants in the posterior region |
| Stefanelli et al., 2020 | PROMs or CROMs were not evaluated |
| Tang et al., 2018 | The article is not in English. |
| Vercruyssen et al., 2014 | The study aimed to compare guided and conventional surgery for implant placement |
| Xie et al., 2024 | PROMs or CROMs were not evaluated |
| Xue et al., 2023 | Implant placement in partially edentulous patients |

**References**

1. Aboelez, M. A., Elezz, M. G. A., Abdraboh, A. E., & Elsyad, M. A. (2022). Angled ball and locator attachments for immediate loaded inclined implants used to retain maxillary overdentures: A cross over study of patient satisfaction and oral health related quality of life. Clinical implant dentistry and related research, 24(3), 391–400. <https://doi.org/10.1111/cid.13089>
2. Aboelez, M. A., Ibrahim, C. R. M., Helmy, M. H. E., & Elsyad, M. A. (2024). Vertical versus angled immediately loaded implants for assisting maxillary overdentures with locator attachments: A preliminary results of one-year randomized clinical trial. Clinical implant dentistry and related research, 26(1), 103–112. <https://doi.org/10.1111/cid.13291>
3. Agliardi, E. L., Pozzi, A., Stappert, C. F., Benzi, R., Romeo, D., & Gherlone, E. (2014). Immediate fixed rehabilitation of the edentulous maxilla: a prospective clinical and radiological study after 3 years of loading. Clinical implant dentistry and related research, 16(2), 292–302. <https://doi.org/10.1111/j.1708-8208.2012.00482.x>
4. Agliardi, E. L., Romeo, D., Panigatti, S., de Araújo Nobre, M., & Maló, P. (2017). Immediate full-arch rehabilitation of the severely atrophic maxilla supported by zygomatic implants: a prospective clinical study with minimum follow-up of 6 years. International journal of oral and maxillofacial surgery, 46(12), 1592–1599. <https://doi.org/10.1016/j.ijom.2017.05.023>
5. Amorfini, L., Migliorati, M., Drago, S., & Silvestrini-Biavati, A. (2017). Immediately Loaded Implants in Rehabilitation of the Maxilla: A Two-Year Randomized Clinical Trial of Guided Surgery versus Standard Procedure. Clinical implant dentistry and related research, 19(2), 280–295. <https://doi.org/10.1111/cid.12459>
6. Anuradha, M., Babaji, H. V., Hiremath, N. V., Usha, V. A., Kumar, A., Nandkeoliar, T., & Verma, S. (2020). Assessment of basal implants in compromised ridges. Journal of family medicine and primary care, 9(4), 2067–2070. <https://doi.org/10.4103/jfmpc.jfmpc_1149_19>
7. Balaguer, J., Ata-Ali, J., Peñarrocha-Oltra, D., García, B., & Peñarrocha-Diago, M. (2015). Long-term survival rates of implants supporting overdentures. The Journal of oral implantology, 41(2), 173–177. <https://doi.org/10.1563/AAID-JOI-D-12-00178>
8. Bernard, L., Vercruyssen, M., Vanderveken, J., Coucke, W., Quirynen, M., & Naert, I. (2019). Randomized controlled trial comparing immediate loading with conventional loading using cone-anchored implant-supported screw-retained removable prostheses: A 2-year follow-up clinical trial. The Journal of prosthetic dentistry, 121(2), 258–264. <https://doi.org/10.1016/j.prosdent.2018.03.022>
9. Bettach, R., Taschieri, S., Boukhris, G., & Del Fabbro, M. (2015). Implant survival after preparation of the implant site using a single bur: a case series. Clinical implant dentistry and related research, 17(1), 13–21. <https://doi.org/10.1111/cid.12082>
10. Browaeys, H., Dierens, M., Ruyffelaert, C., Matthijs, C., De Bruyn, H., & Vandeweghe, S. (2015). Ongoing Crestal Bone Loss around Implants Subjected to Computer-Guided Flapless Surgery and Immediate Loading Using the All-on-4® Concept. Clinical implant dentistry and related research, 17(5), 831–843. <https://doi.org/10.1111/cid.12197>
11. Busenlechner, D., Mailath-Pokorny, G., Haas, R., Fürhauser, R., Eder, C., Pommer, B., & Watzek, G. (2016). Graftless Full-Arch Implant Rehabilitation with Interantral Implants and Immediate or Delayed Loading-Part II: Transition from the Failing Maxillary Dentition. The International journal of oral & maxillofacial implants, 31(5), 1150–1155. <https://doi.org/10.11607/jomi.4326>
12. Caramês, J. M. M., Vieira, F. A., Caramês, G. B., Pinto, A. C., Francisco, H. C. O., & Marques, D. N. D. S. (2022). Guided Bone Regeneration in the Edentulous Atrophic Maxilla Using Deproteinized Bovine Bone Mineral (DBBM) Combined with Platelet-Rich Fibrin (PRF)-A Prospective Study. Journal of clinical medicine, 11(3), 894. <https://doi.org/10.3390/jcm11030894>
13. Chen, Y., Yuan, S., Zhou, N., & Man, Y. (2017). Transcrestal sinus floor augmentation with immediate implant placement applied in three types of fresh extraction sockets: A clinical prospective study with 1-year follow-up. Clinical implant dentistry and related research, 19(6), 1034–1043. <https://doi.org/10.1111/cid.12529>
14. Davó, R., Felice, P., Pistilli, R., Barausse, C., Marti-Pages, C., Ferrer-Fuertes, A., Ippolito, D. R., & Esposito, M. (2018). Immediately loaded zygomatic implants vs conventional dental implants in augmented atrophic maxillae: 1-year post-loading results from a multicentre randomised controlled trial. European journal of oral implantology, 11(2), 145–161.
15. Ebinger, A., Katsoulis, J., Hakimi, M., Mazzi, D., & Mericske-Stern, R. (2016). Mucosal Manifestations in the Edentulous Maxilla with Implant Supported Prostheses: Clinical Results from a Well-Maintained Patient Cohort. Clinical implant dentistry and related research, 18(4), 639–648. <https://doi.org/10.1111/cid.12345>
16. Fayek, N. H., Mahrous, A. I., Shaaban, A. A. E., & ELsyad, M. A. (2022). Patient Satisfaction and Prosthetic Complications of Maxillary Implant Overdentures Opposing Mandibular Implant Overdentures with Bar, Telescopic, and Stud Attachments: A 1-Year Prospective Trial. The International journal of oral & maxillofacial implants, 37(5), 1044–1054. <https://doi.org/10.11607/jomi.9610>
17. Felice, P., Soardi, E., Pellegrino, G., Pistilli, R., Marchetti, C., Gessaroli, M., & Esposito, M. (2011). Treatment of the atrophic edentulous maxilla: short implants versus bone augmentation for placing longer implants. Five-month post-loading results of a pilot randomised controlled trial. European journal of oral implantology, 4(3), 191–202.
18. Fernandes, D., Marques, T., Borges, T., & Montero, J. (2023). Volumetric analysis on the use of customized healing abutments with or without connective tissue graft at flapless maxillary immediate implant placement: A randomized clinical trial. Clinical oral implants research, 34(9), 934–946. <https://doi.org/10.1111/clr.14119>
19. Fernández-Ruiz, J. A., Sánchez-Siles, M., Guerrero-Sánchez, Y., Pato-Mourelo, J., & Camacho-Alonso, F. (2021). Evaluation of Quality of Life and Satisfaction in Patients with Fixed Prostheses on Zygomatic Implants Compared with the All-on-Four Concept: A Prospective Randomized Clinical Study. International journal of environmental research and public health, 18(7), 3426. <https://doi.org/10.3390/ijerph18073426>
20. Mañes Ferrer, J. F., Fernández-Estevan, L., Selva-Otaolaurruchi, E., Labaig-Rueda, C., Solá-Ruíz, M. F., & Agustín-Panadero, R. (2020). Maxillary Implant-Supported Overdentures: Mechanical Behavior Comparing Individual Axial and Bar Retention Systems. A Cohort Study of Edentulous Patients. Medicina (Kaunas, Lithuania), 56(3), 139. <https://doi.org/10.3390/medicina56030139>
21. Gong, Z., Lin, Y., & Di, P. (2023). Plaque accumulation on the fitting surface of full-arch implant-supported fixed prostheses with contact or noncontact pontics: A split mouth randomized controlled trial. Journal of esthetic and restorative dentistry : official publication of the American Academy of Esthetic Dentistry ... [et al.], 35(7), 1077–1084. <https://doi.org/10.1111/jerd.13062>
22. Kuoppala, R., & Raustia, A. (2015). Preliminary Observations Regarding Treatment Outcomes in Patients Treated with Maxillary Implant Overdentures in a University Clinic. The International journal of prosthodontics, 28(6), 637–640. <https://doi.org/10.11607/ijp.4384>
23. Li, S., Di, P., Zhang, Y., & Lin, Y. (2017). Immediate implant and rehabilitation based on All-on-4 concept in patients with generalized aggressive periodontitis: A medium-term prospective study. Clinical implant dentistry and related research, 19(3), 559–571. <https://doi.org/10.1111/cid.12483>
24. Lie, S. A. N., Speksnijder, C. M., Kalic, H., & Kessler, P. A. W. H. (2024). Masticatory function in edentulous patients wearing implant overdentures after graftless maxillary sinus membrane elevation. Journal of oral rehabilitation, 51(6), 1005–1015. <https://doi.org/10.1111/joor.13675>
25. Marković, A., Mišić, T., Janjić, B., Šćepanović, M., Trifković, B., Ilić, B., Todorović, A. M., Marković, J., & Dard, M. M. (2022). Immediate Vs Early Loading of Bone Level Tapered Dental Implants With Hydrophilic Surface in Rehabilitation of Fully Edentulous Maxilla: Clinical and Patient Centered Outcomes. The Journal of oral implantology, 48(5), 358–369. <https://doi.org/10.1563/aaid-joi-D-21-00045>
26. Meloni, S. M., Tallarico, M., Pisano, M., Xhanari, E., & Canullo, L. (2017). Immediate Loading of Fixed Complete Denture Prosthesis Supported by 4-8 Implants Placed Using Guided Surgery: A 5-Year Prospective Study on 66 Patients with 356 Implants. Clinical implant dentistry and related research, 19(1), 195–206. <https://doi.org/10.1111/cid.12449>
27. Mendes, F. A., Borges, T. F., Gonçalves, L. C., de Oliveira, T. R., do Prado, C. J., & das Neves, F. D. (2016). Effects of new implant-retained overdentures on masticatory function, satisfaction and quality of life. Efeito de novas overdentures implanto retidas na função mastigatória, satisfação e qualidade de vida. Acta odontologica latinoamericana : AOL, 29(2), 123–129.
28. Niedermaier, R., Stelzle, F., Riemann, M., Bolz, W., Schuh, P., & Wachtel, H. (2017). Implant-Supported Immediately Loaded Fixed Full-Arch Dentures: Evaluation of Implant Survival Rates in a Case Cohort of up to 7 Years. Clinical implant dentistry and related research, 19(1), 4–19. <https://doi.org/10.1111/cid.12421>
29. Nomiyama, L. M., Matumoto, E. K., Corrêa, M. G., Cirano, F. R., Ribeiro, F. V., Pimentel, S. P., & Casati, M. Z. (2023). Comparison between flapless-guided and conventional surgery for implant placement: a 12-month randomized clinical trial. Clinical oral investigations, 27(4), 1665–1679. <https://doi.org/10.1007/s00784-022-04793-3>
30. Onclin, P., Speksnijder, C. M., Vissink, A., Meijer, H. J. A., & Raghoebar, G. M. (2023). Two or four implants for maxillary overdentures in edentulous patients: 1-year results of a randomized controlled trial. Clinical implant dentistry and related research, 25(6), 1138–1148. <https://doi.org/10.1111/cid.13262>
31. Osman, R. B., Morgaine, K. C., Duncan, W., Swain, M. V., & Ma, S. (2014). Patients' perspectives on zirconia and titanium implants with a novel distribution supporting maxillary and mandibular overdentures: a qualitative study. Clinical oral implants research, 25(5), 587–597. <https://doi.org/10.1111/clr.12106>
32. Papaspyridakos, P., & Chronopoulos, V. (2014). Transition from failing dentition to complete-arch implant rehabilitation with a staged approach: a 3-year clinical report. The Journal of prosthetic dentistry, 112(3), 423–428. <https://doi.org/10.1016/j.prosdent.2014.01.003>
33. Peñarrocha-Oltra, D., Peñarrocha-Diago, M., Canullo, L., Covani, U., & Peñarrocha, M. (2014). Patient-reported outcomes of immediate versus conventional loading with fixed full-arch prostheses in the maxilla: a nonrandomized controlled prospective study. The International journal of oral & maxillofacial implants, 29(3), 690–698. <https://doi.org/10.11607/jomi.3516>
34. Rignon-Bret, C., Wulfman, C., Valet, F., Hadida, A., Nguyen, T. H., Aidan, A., & Naveau, A. (2022). Radiographic evaluation of a bone substitute material in alveolar ridge preservation for maxillary removable immediate dentures: A randomized controlled trial. The Journal of prosthetic dentistry, 128(5), 928–935. <https://doi.org/10.1016/j.prosdent.2021.02.013>
35. Rossi, F., Tuci, L., Ferraioli, L., Ricci, E., Suerica, A., Botticelli, D., Pellegrino, G., & Felice, P. (2021). Two-Year Follow-Up of 4-mm-Long Implants Used as Distal Support of Full-Arch FDPs Compared to 10-mm Implants Installed after Sinus Floor Elevation. A Randomized Clinical Trial. International journal of environmental research and public health, 18(7), 3846. <https://doi.org/10.3390/ijerph18073846>
36. Slot, W., Raghoebar, G. M., Vissink, A., & Meijer, H. J. (2014). Maxillary overdentures supported by anteriorly or posteriorly placed implants opposed by a natural dentition in the mandible: a 1-year prospective case series study. Clinical implant dentistry and related research, 16(1), 51–61. <https://doi.org/10.1111/j.1708-8208.2012.00459.x>
37. Slot, W., Raghoebar, G. M., Cune, M. S., Vissink, A., & Meijer, H. J. A. (2023). Maxillary overdentures supported by four or six implants in the anterior region: 10-year randomized controlled trial results. Journal of clinical periodontology, 50(1), 36–44. <https://doi.org/10.1111/jcpe.13726>
38. Slot, W., Raghoebar, G. M., Cune, M. S., Vissink, A., & Meijer, H. J. A. (2022). Maxillary bar overdentures on four or six posterior implants: 10-year results from a randomized clinical trial. Clinical oral implants research, 33(11), 1147–1156. <https://doi.org/10.1111/clr.13997>
39. Stefanelli, L. V., Mandelaris, G. A., Franchina, A., Pranno, N., Pagliarulo, M., Cera, F., Maltese, F., Angelis, F., & Carlo, S. D. (2020). Accuracy of Dynamic Navigation System Workflow for Implant Supported Full Arch Prosthesis: A Case Series. International journal of environmental research and public health, 17(14), 5038. <https://doi.org/10.3390/ijerph17145038>
40. Tang, T., Zhang, L., Hou, Y. F., Jiang, Y. C., Wang, X. D., & Chen, N. (2018). Shanghai kou qiang yi xue = Shanghai journal of stomatology, 27(1), 52–55.
41. Vercruyssen, M., van de Wiele, G., Teughels, W., Naert, I., Jacobs, R., & Quirynen, M. (2014). Implant- and patient-centred outcomes of guided surgery, a 1-year follow-up: An RCT comparing guided surgery with conventional implant placement. Journal of clinical periodontology, 41(12), 1154–1160. <https://doi.org/10.1111/jcpe.12305>
42. Xie, R., Liu, Y., Wei, H., Zhang, T., Bai, S., & Zhao, Y. (2024). Clinical evaluation of autonomous robotic-assisted full-arch implant surgery: A 1-year prospective clinical study. Clinical oral implants research, 35(4), 443–453. <https://doi.org/10.1111/clr.14243>
43. Xue, H., Wen, J., Liu, C., Shuai, X., Zhang, X., & Kang, N. (2023). Modified transcrestal sinus floor elevation with concomitant implant placement in edentulous posterior maxillae with residual bone height of 5 mm or less: a non-controlled prospective study. International journal of oral and maxillofacial surgery, 52(4), 495–502. <https://doi.org/10.1016/j.ijom.2022.08.014>
