# Supplementary material for: Patient‐ and Clinician‐Reported Outcomes and Outcome Measures Evaluating Timing of Implant Placement in the Edentulous Maxilla: A Systematic Review of Clinical Studies
Source: Clin Oral Implants Res. 2026 Feb 24;37(Suppl 30):S302–31. doi: 10.1111/clr.14454 (PMC12930140; doi:10.1111/clr.14454)
Supplement: Supplementary file 3 — Table S3. References for the methods used in the studies. [file CLR-37-S302-s003.docx]

Table S3 - References for the methods used in the studies

| **Study** | **PROs/PROMs** | **References for PROs/PROMs cited in the study** | **General validation** | **Validation in the context of implant treatment** |
| --- | --- | --- | --- | --- |
| Bouhy et al., 2023 | OHIP-20 (OHIP-EDENT) | 1. Allen, F., & Locker, D. (2002). A modified short version of the oral health impact profile for assessing health-related quality of life in edentulous adults. The International journal of prosthodontics, 15(5), 446–450. | Yes | Yes |
|  | Patient satisfaction (an adaptation of the McGill Denture Satisfaction) | 1. Awad, M. A., & Feine, J. S. (1998). Measuring patient satisfaction with mandibular prostheses. Community Dentistry and Oral Epidemiology, 26(6), 400–405. 2. Awad, M. A., Lund, J. P., Dufresne, E., & Feine, J. S. (2003). Comparing the efficacy of mandibular implant-retained overdentures and conventional dentures among middle-aged edentulous patients: Satisfaction and functional assessment. The International Journal of Prosthodontics, 16(2), 117–122. 3. de Grandmont, P., Feine, J. S., Tache, R., Boudrias, P., Donohue, W. B., Tanguay, R., & Lund, J. P. (1994). Within-subject comparisons of implant-supported mandibular prostheses: Psychometric evaluation. Journal of Dental Research, 73(5), 1096–1104. 4. Michaud, P. L., de Grandmont, P., Feine, J. S., & Emami, E. (2012). Measuring patient-based outcomes: Is treatment satisfaction associated with oral health-related quality of life? Journal of Dentistry, 40(8), 624–631. | Yes | Yes |
| Boven et al., 2017 | Functional complaints about the upper denture | 1. Vervoorn, J. M., Duinkerke, A. S., Luteijn, F., & van de Poel, A. C. (1988). Assessment of denture satisfaction. Community dentistry and oral epidemiology, 16(6), 364–367. | Yes | No |
|  | Patients’ eating ability with their upper denture | 1. Stellingsma, K., Slagter, A. P., Stegenga, B., Raghoebar, G. M., & Meijer, H. J. (2005). Masticatory function in patients with an extremely resorbed mandible restored with mandibular implant-retained overdentures: comparison of three types of treatment protocols. Journal of oral rehabilitation, 32(6), 403–410. | No | No |
|  | Patients’ overall treatment satisfaction (10-point rating scale) | None | No | No |
| Erkapers et al., 2017 | OHIP-49 | 1. Slade, G. D., & Spencer, A. J. (1994). Development and evaluation of the Oral Health Impact Profile. Community dental health, 11(1), 3–11. 2. Awad, M. A., Locker, D., Korner-Bitensky, N., & Feine, J. S. (2000). Measuring the effect of intra-oral implant rehabilitation on health-related quality of life in a randomized controlled clinical trial. Journal of dental research, 79(9), 1659–1663. 3. Allen, F., & Locker, D. (2002). A modified short version of the oral health impact profile for assessing health-related quality of life in edentulous adults. The International journal of prosthodontics, 15(5), 446–450. 4. Allen, P. F., McMillan, A. S., Walshaw, D., & Locker, D. (1999). A comparison of the validity of generic- and disease-specific measures in the assessment of oral health-related quality of life. Community dentistry and oral epidemiology, 27(5), 344–352. 5. Slade G. D. (1998). Assessing change in quality of life using the Oral Health Impact Profile. Community dentistry and oral epidemiology, 26(1), 52–61. 6. Larsson, P., List, T., Lundström, I., Marcusson, A., & Ohrbach, R. (2004). Reliability and validity of a Swedish version of the Oral Health Impact Profile (OHIP-S). Acta odontologica Scandinavica, 62(3), 147–152. | Yes | No |
| Fonteyne et al., 2019 | Articulation | 1. Van Lierde, K. M., Corthals, P., Browaeys, H., Mussche, P., Van Kerckhove, E., & De Bruyn, H. (2011). Impact of anterior single-tooth implants on quality of life, articulation and oromyofunctional behaviour: a pilot study. Journal of oral rehabilitation, 38(3), 170–175. 2. Van Borsel J. (1996). Articulation in Down's syndrome adolescents and adults. European journal of disorders of communication : the journal of the College of Speech and Language Therapists, London, 31(4), 415–444. | No | No |
|  | Oromyofunctional behaviour | 1. Van Lierde, K. M., Corthals, P., Browaeys, H., Mussche, P., Van Kerckhove, E., & De Bruyn, H. (2011). Impact of anterior single-tooth implants on quality of life, articulation and oromyofunctional behaviour: a pilot study. Journal of oral rehabilitation, 38(3), 170–175. 2. Lembrechts, D., Verschueren, D., Heulens, H., Valkenburg, H. A., & Feenstra, L. (1999). Effect of a logopedic instruction program after adenoidectomy on open mouth posture: a single-blind study. Folia phoniatrica et logopaedica : official organ of the International Association of Logopedics and Phoniatrics (IALP), 51(3), 117–123. | No | No |
|  | OHIP-14 | 1. Slade, G.D. (1997) Derivation and validation of a short-form oral health impact profile. Community Dentistry and Oral Epidemiology 25: 284–290. | Yes | No |
|  | Speech | 1. Van Lierde, K. M., Corthals, P., Browaeys, H., Mussche, P., Van Kerckhove, E., & De Bruyn, H. (2011). Impact of anterior single-tooth implants on quality of life, articulation and oromyofunctional behaviour: a pilot study. Journal of oral rehabilitation, 38(3), 170–175. | No | No |
|  | Satisfaction with speech (using the domain "functional limitation" of OHIP-14 | 1. Slade, G. D., & Spencer, A. J. (1994). Development and evaluation of the Oral Health Impact Profile. Community dental health, 11(1), 3–11. | No | No |
|  | Satisfaction with oral health (10 cm visual analogue scale) | None | No | No |
|  | Satisfaction with speech (10 cm visual analogue scale) | None | No | No |
| Furhauser et al., 2016 | Pain intensity | None | No | No |
|  | Swelling | None | No | No |
|  | Postoperative impairment of everyday life and work | None | No | No |
| Menini et al., 2016 | Swelling | None | No | No |
|  | Pain | Breivik, H., Borchgrevink, P. C., Allen, S. M., Rosseland, L. A., Romundstad, L., Hals, E. K., Kvarstein, G., & Stubhaug, A. (2008). Assessment of pain. British journal of anaesthesia, 101(1), 17–24. | No | No |
|  | Psychosocial impact (comfort) | Paice, J. A., & Cohen, F. L. (1997). Validity of a verbally administered numeric rating scale to measure cancer pain intensity. Cancer nursing, 20(2), 88–93. | Yes | No |
|  | Postoperative drug administration | None | No | No |
| Misumi et al., 2014 | OHIP-14 | 1. Yamazaki, M., Inukai, M., Baba, K. & John, M.T. (2007) Japanese version of the Oral Health Impact Profile (OHIP-J). Journal of Oral Rehabilitation 34: 159–168. 2. Baba, K., Inukai, M. & John, M.T. (2008) Feasibility of oral health-related quality of life assessment in prosthodontic patients using abbreviated Oral Health Impact Profile questionnaires. Journal of Oral Rehabilitation 35: 224–228. 3. Slade, G.D. (1997) Derivation and validation of a short-form oral health impact profile. Community Dentistry and Oral Epidemiology 25: 284–290. | Yes | No |
| Van Doorne et al., 2020 | Pain | 1. Griffin, T. J., Cheung, W. S., Zavras, A. I., & Damoulis, P. D. (2006). Postoperative complications following gingival augmentation procedures. Journal of periodontology, 77(12), 2070–2079.  2. Mei, C. C., Lee, F. Y., & Yeh, H. C. (2016). Assessment of pain perception following periodontal and implant surgeries. Journal of clinical periodontology, 43(12), 1151–1159. | No | No |
|  | Patient satisfaction | None | No | No |
| Pomares-puig et al., 2023 | Patient wellbeing during the surgical procedure | None | No | No |
|  | Intraoperative pain. | None | No | No |
|  | Satisfaction during the surgery. | None | No | No |
|  | OHIP-14 (Spanish version) | 1. Montero-Martín, J., Bravo-Pérez, M., Albaladejo-Martínez, A., Hernández-Martín, L. A., & Rosel-Gallardo, E. M. (2009). Validation the Oral Health Impact Profile (OHIP-14sp) for adults in Spain. Medicina oral, patologia oral y cirugia bucal, 14(1), E44–E50. | Yes | No |
|  | Intake of analgesic and anti-inflammatory medication. | None | No | No |
|  | Postoperative pain | None | No | No |
| Testori et al., 2021 | Postsurgery quality of life | None | No | No |
|  | Postprosthetic quality of life | None | No | No |
| Yamada et al., 2015 | OHIP-J49 | 1. Slade, G. D., & Spencer, A. J. (1994). Development and evaluation of the Oral Health Impact Profile. Community dental health, 11(1), 3–11. 2. Yamazaki, M., Inukai, M., Baba, K., & John, M. T. (2007). Japanese version of the Oral Health Impact Profile (OHIP-J). Journal of oral rehabilitation, 34(3), 159–168. | Yes | No |
|  | Postoperative pain | None | No | No |
|  | Postoperative swelling | 1. Arisan, V., Karabuda, C. Z., & Ozdemir, T. (2010). Implant surgery using bone- and mucosa-supported stereolithographic guides in totally edentulous jaws: surgical and post-operative outcomes of computer-aided vs. standard techniques. Clinical oral implants research, 21(9), 980–988. | No | No |
| Zembic et al., 2019 | OHIP-EDENT | 1. Heydecke, G., Klemetti, E., Awad, M. A., Lund, J. P., & Feine, J. S. (2003). Relationship between prosthodontic evaluation and patient ratings of mandibular conventional and implant prostheses. The International journal of prosthodontics, 16(3), 307–312. 2. Allen, F., & Locker, D. (2002). A modified short version of the oral health impact profile for assessing health-related quality of life in edentulous adults. The International journal of prosthodontics, 15(5), 446–450. | Yes | Yes |
|  | Patient satisfaction | 1. de Grandmont, P., Feine, J. S., Taché, R., Boudrias, P., Donohue, W. B., Tanguay, R., & Lund, J. P. (1994). Within-subject comparisons of implant-supported mandibular prostheses: psychometric evaluation. Journal of dental research, 73(5), 1096–1104. | No | No |
| Zhang et al., 2016 | Patient satisfaction | None | No | No |
| **Study** | **CROs/CROMs** | **References for CROs/CROMs cited in the study** | | |
| Bouhy et al., 2023 | Prosthodontic outcomes – Prosthetic complications | None | | |
|  | Prosthodontic success | None | | |
|  | Accepted prosthodontic maintenance events | None | | |
|  | Prosthesis failure | None | | |
|  | Implant survival | None | | |
|  | Sulcular Modified Bleeding Index | Mombelli, A., van Oosten, M. A., Schurch, E., Jr, & Land, N. P. (1987). The microbiota associated with successful or failing osseointegrated titanium implants. *Oral microbiology and immunology*, *2*(4), 145–151. | | |
|  | Plaque index | Loe, H., & Silness, J. (1963). Periodontal disease in pregnancy. I. Prevalence and severity. *Acta odontologica Scandinavica*, *21*, 533–551. | | |
|  | Probing depth | None | | |
| Boven et al., 2017 | Implant survival | None | | |
|  | Peri-implant bone level changes | None | | |
|  | Modified plaque index | Mombelli, A., van Oosten, M. A., Schurch, E., Jr, & Land, N. P. (1987). The microbiota associated with successful or failing osseointegrated titanium implants. *Oral microbiology and immunology*, *2*(4), 145–151. | | |
|  | Presence of calculus | Mombelli, A., van Oosten, M. A., Schurch, E., Jr, & Land, N. P. (1987). The microbiota associated with successful or failing osseointegrated titanium implants. *Oral microbiology and immunology*, *2*(4), 145–151. | | |
|  | Peri-implant inflammation | Loe, H., & Silness, J. (1963). Periodontal disease in pregnancy. I. Prevalence and severity. *Acta odontologica Scandinavica*, *21*, 533–551. | | |
|  | Sulcular Modified Bleeding Index | Mombelli, A., van Oosten, M. A., Schurch, E., Jr, & Land, N. P. (1987). The microbiota associated with successful or failing osseointegrated titanium implants. *Oral microbiology and immunology*, *2*(4), 145–151. | | |
|  | Probing depth | None | | |
| Erkapers et al., 2017 | Prosthetic complications | None | | |
| Fonteyne et al., 2019 | Articulation | None | | |
|  | Oromyofunctional behaviour. | None | | |
| Menini et al., 2016 | Swelling | None | | |
| Pomares-puig et al., 2023 | Accuracy variables (implant position deviation) | None | | |
| Testori et al., 2021 | Implant survival | None | | |
|  | Prosthetic survival | None | | |
| Van Doorne et al., 2020 | Implant survival | None | | |
|  | Prosthetic survival | None | | |
| Yamada et al., 2015 | Insertion torque | None | | |
|  | Surgical time | None | | |
|  | Implant survival | D’haese J, Vervaeke S, Verbanck N, De Bruyn H. Clinical and  radiographic outcome of implants placed using stereolithographic  guided surgery: A prospective monocenter study. Int J Oral Maxillofac Implants 2013;28:205–215. | | |
|  | Prosthetic survival | None | | |
|  | Peri-implant bone level changes | None | | |
|  | Post operative swelling | Arisan, V., Karabuda, C. Z., & Ozdemir, T. (2010). Implant surgery using bone- and mucosa-supported stereolithographic guides in totally edentulous jaws: surgical and post-operative outcomes of computer-aided vs. standard techniques. *Clinical oral implants research*, *21*(9), 980–988. https://doi.org/10.1111/j.1600-0501.2010.01957.x | | |
|  | Complications (surgical, with the provisional and postoperative) | None | | |
| Zhang et al., 2016 | Implant success | 1. Buser D, Mericske-Stern R, Bernard JP, Behneke A,   Behneke N, Hirt HP, Belser UC, Lang NP. Long-term  evaluation of non-submerged ITI implants. Part 1: 8-year  life table analysis of a prospective multi-center study with  2359 implants. Clin Oral Implants Res 1997; 8:161–172.   1. Cochran DL, Buser D, ten Bruggenkate CM, et al. The   use of reduced healing times on ITI implants with a sandblasted and acid-etched (SLA) surface: early results from  clinical trials on ITI SLA implants. Clin Oral Implants  Res 2002; 13:144–153. | | |
|  | Prosthetic success | None | | |
|  | Prosthetic - Technical complications | Zollner A, Belser U, Working G. Factors influencing survival of reconstructions. Consensus report of Working Group 2. Clin Oral Implants Res 2007; 18(Suppl 3):114–116. | | |
|  | Modified plaque index | Mombelli A, Van Oosten MAC, Schurch JE, Lang NP. €  The microbiota associated with successful or failing  osseointegrated titanium implants. Oral Microbiol Immunol 1987; 2:145–151 | | |
|  | Probing depth | Mombelli A, Van Oosten MAC, Schurch JE, Lang NP. €  The microbiota associated with successful or failing  osseointegrated titanium implants. Oral Microbiol Immunol 1987; 2:145–151 | | |
|  | Modified bleeding index | Mombelli A, Van Oosten MAC, Schurch JE, Lang NP. €  The microbiota associated with successful or failing  osseointegrated titanium implants. Oral Microbiol Immunol 1987; 2:145–151 | | |
|  | Peri-implant bone level changes – marginal bone loss | None | | |
|  | Peri-implantitis | Padial-Molina M, Suarez F, Rios HF, Galindo-Moreno P,  Wang HL. Guidelines for the diagnosis and treatment of  peri-implant diseases. Int J Periodontics Restorative Dent  2014; 34:e102–e111. | | |
